# Molecular characteristics of breast tumors in patients screened for germline predisposition from a population-based observational study

**DOI:** 10.1186/s13073-023-01177-4

**Published:** 2023-04-14

**Authors:** Deborah F. Nacer, Johan Vallon-Christersson, Nicklas Nordborg, Hans Ehrencrona, Anders Kvist, Åke Borg, Johan Staaf

**Affiliations:** 1 Division of Oncology, Department of Clinical Sciences Lund, Lund University, Lund, Sweden; 2 Division of Translational Cancer Research, Department of Laboratory Medicine, Lund University, Medicon Village, Lund, SE-22381 Sweden; 3 Department of Genetics and Pathology, Laboratory Medicine, Region Skåne, Lund, Sweden; 4 Division of Clinical Genetics, Department of Laboratory Medicine, Lund University, Lund, Sweden

**Keywords:** Hereditary breast cancer, Clinical screening, Gene expression, Gene variants, Molecular subtypes

## Abstract

**Background:**

Pathogenic germline variants (PGVs) in certain genes are linked to higher lifetime risk of developing breast cancer and can influence preventive surgery decisions and therapy choices. Public health programs offer genetic screening based on criteria designed to assess personal risk and identify individuals more likely to carry PGVs, dividing patients into screened and non-screened groups. How tumor biology and clinicopathological characteristics differ between these groups is understudied and could guide refinement of screening criteria.

**Methods:**

Six thousand six hundred sixty breast cancer patients diagnosed in South Sweden during 2010–2018 were included with available clinicopathological and RNA sequencing data, 900 (13.5%) of which had genes screened for PGVs through routine clinical screening programs. We compared characteristics of screened patients and tumors to non-screened patients, as well as between screened patients with (*n* = 124) and without (*n* = 776) PGVs.

**Results:**

Broadly, breast tumors in screened patients showed features of a more aggressive disease. However, few differences related to tumor biology or patient outcome remained significant after stratification by clinical subgroups or PAM50 subtypes. Triple-negative breast cancer (TNBC), the subgroup most enriched for PGVs, showed the most differences between screening subpopulations (e.g., higher tumor proliferation in screened cases). Significant differences in PGV prevalence were found between clinical subgroups/molecular subtypes, e.g., TNBC cases were enriched for *BRCA1* PGVs. In general, clinicopathological differences between screened and non-screened patients mimicked those between patients with and without PGVs, e.g., younger age at diagnosis for positive cases. However, differences in tumor biology/microenvironment such as immune cell composition were additionally seen within PGV carriers/non-carriers in ER + /HER2 − cases, but not between screening subpopulations in this subgroup.

**Conclusions:**

Characterization of molecular tumor features in patients clinically screened and not screened for PGVs represents a relevant read-out of guideline criteria. The general lack of molecular differences between screened/non-screened patients after stratification by relevant breast cancer subsets questions the ability to improve the identification of screening candidates based on currently used patient and tumor characteristics, pointing us towards universal screening. Nevertheless, while that is not attained, molecular differences identified between PGV carriers/non-carriers suggest the possibility of further refining patient selection within certain patient subsets using RNA-seq through, e.g., gene signatures.

**Trial registration:**

The Sweden Cancerome Analysis Network – Breast (SCAN-B) was prospectively registered at ClinicalTrials.gov under the identifier NCT02306096.

**Supplementary Information:**

The online version contains supplementary material available at 10.1186/s13073-023-01177-4.

## Background

Breast cancer is the most common malignancy in women. Underlying causes of disease include lifestyle factors and reproductive history, but also germline predisposition. Proportions vary with ancestry, but an estimated 5–10% of breast cancer cases carry germline variants in genes associated with moderate to strong breast cancer risk (e.g., *BRCA1*, *BRCA2*, and *CHEK2*) [1, 2]. These variants can influence disease onset, progression, biology of the tumor, potential for therapy, risk of recurrence, and cancer risk of close relatives [3]. This has led to clinical genetic screening of women to detect and monitor families at risk, but also to provide access to risk-reducing prophylactic surgery and in certain instances targeted therapies for developed tumors [4, 5]. Criteria for genetic screening are designed to assess personal risk of developing cancer and likelihood of being a carrier of a pathogenic germline variant (PGV) [6, 7]. Although specific criteria vary between countries, they usually include personal and family history of breast and/or ovarian cancer, young age at disease onset, male breast cancer, and in recent years, specific relevant clinical subgroups. Genetic screening offered to all breast cancer patients or to the general population has also been implemented as part of research studies or in specific populations with high prevalence of founder variants [8, 9]. Recommendations regarding which genes to include in testing also vary, in part as an adaptation to the selection criteria implemented in the screening programs [10, 11].

PGVs in certain breast cancer predisposition genes are more strongly associated with specific clinicopathological characteristics and molecular subtypes of breast cancer [12–20]. Specific examples include association of the *CHEK2* c.1100delC variant with ER-positive disease [16, 17], and *BRCA1*, *BRCA2*, *BARD1*, *BRIP1*, *PALB2*, *RAD51C*, *RAD51D*, and *TP53* variants with increased risks of triple-negative breast cancer (i.e., tumors that are negative for estrogen receptor [ER], for progesterone receptor [PR], and for amplification of the human epidermal growth factor receptor 2/erythroblastic oncogene B [*HER2*/*ERBB2*] gene, TNBC) [18–20]. Moreover, PGVs may induce specific genomic patterns as exemplified by inactivating variants in *BRCA1*, which introduces characteristic genetic alterations in the tumor genome, representing the somatic genetic scars of DNA repair deficiency through defective homologous recombination that manifest in mutational and rearrangement signatures [21, 22].

Many studies have investigated the population-based prevalence and risk of breast cancer from PGVs in breast cancer predisposition genes [1, 2, 23–26]. Fewer studies have been able to describe how such PGVs impact clinicopathological and molecular patterns in population-based breast cancer [13–15, 27, 28]. It has been suggested that risk stratification of women with breast cancer in the general population based on patient and tumor marker features is important to identify women at the highest risk of having germline alterations [1, 13]. Consequently, understanding clinical and molecular features and characteristics of not only PGV carriers, but also between patients currently screened for variants according to guidelines (including those with no findings) and the non-screened population of patients in general appears important, representing a read-out of the current patient selection process. In this study, we particularly aimed to address the latter question through the analysis of a population-based cohort of 6660 breast cancer patients profiled by RNA sequencing (RNA-seq) from one single institution in southern Sweden with matched records of clinical genetic screening data. Specifically, we aimed to contrast patients that have been screened according to actual clinical decision versus patients not screened for germline variants in relevant clinical and molecular subsets of breast cancer to determine if screened patients and/or their tumors are associated with specific clinicopathological or molecular characteristics not clearly stated in the testing guidelines, a study currently not reported at this scale.

## Methods

### Unselected population-based breast cancer cohort

**Fig. 1:**
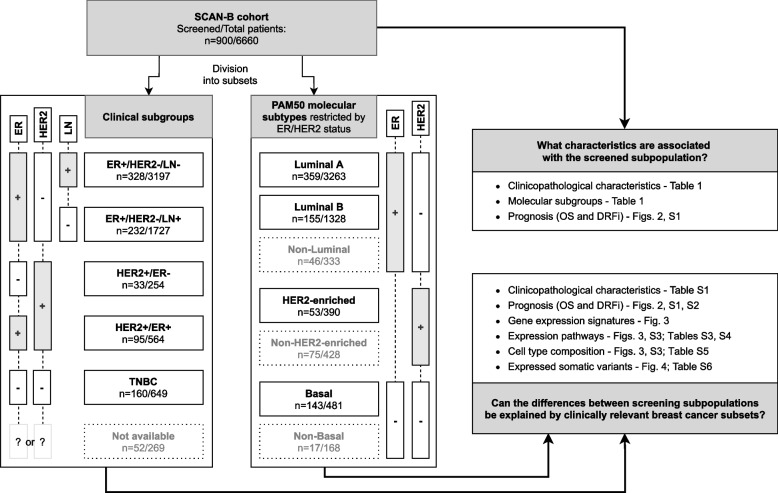
Project outline. Cohort division into clinically relevant breast cancer subgroups and PAM50 molecular subtypes including which information was used to compare the screened and non-screened subpopulations. Patients without ER/HER2 information are not shown in the PAM50 division. Subsets surrounded by dotted lines were not included in the analyses (see Additional file 1: Supplementary Methods)

The Sweden Cancerome Analysis Network – Breast (SCAN-B) initiative [29, 30] (ClinicalTrials.gov ID NCT02306096, prospectively registered) first started enrolling participants in September 2010 and it is still ongoing. It has as primary outcomes the analyses of biomarkers, of their relationship to patient and tumor characteristics, and their relationship with invasive disease-free survival at different time points. In addition, overall survival and breast cancer-specific survival are included as secondary outcomes also calculated at different time points up to a 20-year follow-up. Six thousand six hundred sixty patients diagnosed with primary invasive breast tumors and enrolled in SCAN-B between September 1, 2010, and May 31, 2018, with curated RNA-seq and clinicopathological data available in Staaf et al. [31] were included in this study, a cohort hereafter referred to as SCAN-B. A CONSORT diagram with sample inclusion/exclusion criteria is available in the supplementary material of the original publication [31]. This SCAN-B cohort has been shown to be representative of the underlying healthcare population from which they were recruited [31]. Clinicopathological and molecular subtype information (PAM50 and Risk Of Recurrence [ROR] score [12]) implemented as described in Staaf et al. [31] for the cohort are provided in Table 1. Patient ancestry and race were not available to this study. Patients were divided into clinically relevant subgroups according to ER, PR, and HER2 status (+ = positive, −  = negative), as well as lymph node (LN) status in some cases, resulting in five subgroups (Fig. 1). Subgroup enrollment into SCAN-B was similar between years of the study [31]. For analyses conducted within germline screened patients alone, the five clinical subgroups were combined into only three for larger sample size. Alternatively, patients were divided into four PAM50 molecular subtypes derived from RNA-seq (Fig. 1). Not at all patients could be included in analyses using subgroups/subtypes. See Additional file 1: Supplementary Methods for more information on SCAN-B subsets. Clinicopathological information by subgroup/subtype is provided in Additional file 2: Table S1.

**Table 1:** Clinicopathological characteristics of patients and tumors in SCAN-B divided by screening subpopulation

	**Non-screened patients** (*n* = 5760)	**Screened patients** (*n* = 900)	**Total** (*n* = 6660)	*P*
**Gender**				< 0.001
Female	5732 (99.5%)	880 (97.8%)	6612 (99.3%)	
Male	28 (0.5%)	20 (2.2%)	48 (0.7%)	
**Age at diagnosis (years)**				< 0.001
≤ 40	117 (2.0%)	212 (23.6%)	329 (4.9%)	
41–50	830 (14.4%)	214 (23.8%)	1044 (15.7%)	
51–60	1140 (19.8%)	157 (17.4%)	1297 (19.5%)	
61–70	1850 (32.1%)	205 (22.8%)	2055 (30.9%)	
71–80	1228 (21.3%)	87 (9.7%)	1315 (19.7%)	
≥ 81	595 (10.3%)	25 (2.8%)	620 (9.3%)	
**Nottingham histologic grade (NHG)**				< 0.001
Grade 1	923 (16.0%)	103 (11.4%)	1026 (15.4%)	
Grade 2	2824 (49.0%)	361 (40.1%)	3185 (47.8%)	
Grade 3	1820 (31.6%)	332 (36.9%)	2152 (32.3%)	
Not available	193 (3.4%)	104 (11.6%)	297 (4.5%)	
**Tumor size**				0.68
T1 (< 20 mm)	3329 (57.8%)	547 (60.8%)	3876 (58.2%)	
T2 (20–50 mm)	1743 (30.3%)	267 (29.7%)	2010 (30.2%)	
T3 (> 50 mm)	169 (2.9%)	27 (3.0%)	196 (2.9%)	
Other/Not available	519 (9.0%)	59 (6.6%)	578 (8.7%)	
**Ki67 status**				< 0.001
Low	2890 (50.2%)	341 (37.9%)	3231 (48.5%)	
High	2870 (49.8%)	559 (62.1%)	3429 (51.5%)	
**Risk of recurrence (ROR) score**				< 0.001
Low	1937 (33.6%)	241 (26.8%)	2178 (32.7%)	
Intermediate	1287 (22.3%)	174 (19.3%)	1461 (21.9%)	
High	2329 (40.4%)	433 (48.1%)	2762 (41.5%)	
Not available	207 (3.6%)	52 (5.8%)	259 (3.9%)	
**Clinical subgroup**				< 0.001
ER + /HER2 − /LN −	2869 (49.8%)	328 (36.4%)	3197 (48.0%)	
ER + /HER2 − /LN +	1495 (26.0%)	232 (25.8%)	1727 (25.9%)	
HER2 + /ER −	221 (3.8%)	33 (3.7%)	254 (3.8%)	
HER2 + /ER +	469 (8.1%)	95 (10.6%)	564 (8.5%)	
TNBC	489 (8.5%)	160 (17.8%)	649 (9.7%)	
Not available	217 (3.8%)	52 (5.8%)	269 (4.0%)	
**PAM50 molecular subtype (restricted by ER/HER2 status)**				< 0.001
Luminal A (& ER + /HER2 −)	2904 (50.4%)	359 (39.9%)	3263 (49.0%)	
Luminal B (& ER + /HER2 −)	1173 (20.4%)	155 (17.2%)	1328 (19.9%)	
HER2-enriched (& HER2 +)	337 (5.9%)	53 (5.9%)	390 (5.9%)	
Basal (& ER − /HER2 −)	338 (5.9%)	143 (15.9%)	481 (7.2%)	
Other	1008 (17.5%)	190 (21.1%)	1198 (18.0%)	

*P* chi-square test *p*-value corrected for multiple testing (8 tests) with the Benjamini–Hochberg method. Variable categories “Not available” and “Other” were excluded from calculations

### Screening for variants in predisposition genes

Included SCAN-B patients were cross-matched to clinical genetic screening data performed at the Division of Oncology, Department of Clinical Sciences in Lund, Lund University, Sweden—the main institution for screening in the area. Of 6660 patients, 900 (13.5%) had been referred to counseling and genetic screening according to practitioner’s choice based on at-the-time current guidelines, a clinical decision that is completely separated from enrollment in the SCAN-B study. Current screening criteria rely mainly on patient gender, age, and family history (Table 2), and they were made more inclusive than the previous version [32] by changing, e.g., age cutoffs. Criteria are similar to those proposed in the National Comprehensive Cancer Network (NCCN) Guidelines [7]. The individual cause for a patient’s referral to screening was not accessible to the study due to ethical permissions. Based on clinical screening status, each of the 6660 patients was assigned to either a screened or non-screened subpopulation. Clinicopathological characteristics for the screened and non-screened subpopulation are summarized in Table 1. Most screened patients were analyzed for PGVs in several genes through NGS-based hybrid capture panels [33] that include 11 genes previously associated with breast cancer [1, 2] that are the focus of this study: *ATM*, *BARD1*, *BRCA1*, *BRCA2*, *CDH1*, *CHEK2*, *PALB2*, *PTEN*, *RAD51C*, *RAD51D*, and *TP53*. More information on screening can be found in the Additional file 1: Supplementary Methods. Variants had been classified as benign, likely benign, uncertain, likely pathogenic, or pathogenic, and clinical significance was compared to information on ClinVar [34] at date of accession (May 2020). In this study, likely pathogenic and pathogenic variants are combined and referred to only as pathogenic (similar to [1]), and likely benign and benign variants as benign. Germline variant waterfall plot and variant distribution in specific genes were created with the R packages *ComplexHeatmap* [35] v2.6.2 and *trackViewer* [36] v1.26.2 respectively. Protein domains were obtained from Pfam [37] according to UniProt entries P38398 (BRCA1), P51587 (BRCA2), and O96017 (CHEK2).

**Table 2:** Swedish Breast Cancer Group criteria for screening for variants in predisposition genes as implemented in 2017 and how many patients meet those criteria in SCAN-B. Criteria from before 2017 are available in [32]

**Criteria**	**SCAN-B cases**^b^
Breast cancer ≤ 40 years of age	329
Breast cancer ≤ 50 years of age if there is at least one other case of breast cancer in first-degree or second-degree relatives^a^	NA
Breast cancer ≤ 60 years of age if there are at least two other cases of breast cancer in first-degree or second-degree relatives^a^	NA
Triple-negative breast cancer ≤ 60 years of age	298
Male breast cancer irrespective of age	48
Ovarian cancer including fallopian tube cancer and primary peritoneal carcinomatosis (not mucinous nor borderline) irrespective of age	NA
Meeting criteria for other hereditary syndromes that include breast/ovarian cancer	NA

^a^ The other cancer cases can also be of ovarian cancer, early prostate cancer (before the age of 65), or pancreatic cancer. Bilateral breast cancer counts as two cases.

^b^ *NA* number of patients could not be calculated since family history was not accessible to the study

### Gene expression analyses

RNA sequencing (RNA-seq) data had been obtained from fresh tissue specimens and processed as described [29, 30]. Fragments per kilobase million (FPKM) values were available for all 6660 SCAN-B cases. These were used in several steps such as classifying samples without Ki67 tumor cell proliferation biomarker status into low or high proliferation based on *MKI67* expression values (see Additional file 1: Supplementary Methods). To calculate another tumor proliferation measure, expression values of genes associated with chromosomal instability and cell proliferation rate belonging to the CIN70 signature [38] (Additional file 2: Table S2) were used to estimate rank scores per sample as described in Nacer et al. [39] (see Additional file 1: Supplementary Methods for more details). Here, a low rank score indicates a low in silico level of tumor proliferation for a given sample relative to the cohort. A similar approach was used to calculate an in silico immune response rank score based on 71 highly correlated genes previously associated with this pathway based on a gene network analysis in breast cancer [40] (Additional file 2: Table S2).

Differential gene expression analyses between groups were performed using significance analysis of microarrays (SAM) [41] based on the *samr* R package v3.0 separately for each clinical subgroup and PAM50 subtype with false discovery rate (FDR) adjustment of *p*-values (*p* ≤ 0.01 cut-off, Additional file 1: Supplementary Methods). Whenever up- or downregulated genes were identified in the screened subpopulation, a statistical overrepresentation test was performed using the PANTHER Classification System [42] v17.0 to identify possibly important represented pathways based on gene function as outlined (Additional file 1: Supplementary Methods). To assess potential gene expression variance associated with screening status, dimensionality reduction through Uniform Manifold Approximation and Projection (UMAP) was performed on RNA-seq data for each subset independently with the R packages *tidymodels* v0.1.3 and *embed* v0.1.5 (Additional file 1: Supplementary Methods).

In silico abundance estimates of different cell types were derived from bulk RNA-seq data by two approaches: (i) a marker gene-based method, xCell [43], used to evaluate the presence of 64 cell types locally with the R package v1.1.0, and (ii) a deconvolution-based method, CIBERSORTx [44], used online on absolute mode to impute fractions of six cell types (batch correction and quantile normalization disabled, 100 permutations). Other details and statistical approaches are outlined in Additional file 1: Supplementary Methods.

RNA-seq data were also used to identify expressed somatic variants using 6614 available variant calling files from SCAN-B patients, 893 of which belonged to screened patients. Variant calls were created using a pipeline originally reported by Brueffer et al. [45] but with a few modifications (Additional file 1: Supplementary Methods). Variant calling from RNA has shortcomings when compared to from DNA, but it can still generate similar, valid results (see [45] for a discussion). The pipeline also reports predictions of which genes are affected by the variant and functional impacts it can have. A total of 361,034 variants in nearly 14,500 genes were found in our cohort: 349,965 single-nucleotide variants (SNVs, 97%), 2538 insertions, and 8531 deletions. Most variants were predicted to affect untranslated regions (49.8%) or to cause missense amino acid changes in proteins (33.5%), but several synonymous changes were also identified (11.9%).

### Survival analyses and statistical methods

Survival analyses were performed with R v4.0.3 in RStudio using the *survival* v3.2.7 and *survminer* v0.4.8 packages with overall survival (OS) and distant recurrence-free interval (DRFi) as clinical endpoints. Survival curves were compared using Kaplan–Meier estimates and the log-rank test. Hazard ratios (HR) and a 95% confidence interval (CI) were calculated either through univariate or multivariate Cox regression using the *coxph* function and verified to fulfill assumptions for proportional hazards. Covariates in the multivariate analysis included patient age at diagnosis, NHG status, tumor size, and lymph node status. The latter one was not included for subgroups already stratified by it (e.g., ER + /HER2 − /LN −). The categories “Other” and “Not available” were excluded from statistical calculations, as well as other categories with less than five representatives in the screened subpopulation. All *p*-values reported are two-sided and were compared to a level of significance of 0.05 unless otherwise specified.

## Results

### What characteristics are associated with the screened subpopulation?

A main aim of this study was to contrast the two screening subpopulations to determine which clinicopathological and molecular characteristics were associated with the screened patient group, whether these were explicitly part of the screening guidelines or not, providing us with a read-out of a real-world patient selection process. An outline of the project is included in Fig. 1. In total, 900 patients (13.5%) of the SCAN-B cohort had been referred to genetic testing (referred to as the screened subpopulation hereon). Notably, not all patients that fulfilled the screening criteria at the time of diagnosis were screened in practice. This study did not have access to family cancer history, but based on criteria including age, gender, and clinical subgroup alone, 211 patients in our cohort would have been screened when applying the guidelines from before 2017. However, only 150 (71%) of these were indeed screened for gene variants and therefore placed in the screened subpopulation in this study. When using the more inclusive criteria implemented in 2017 (Table 2), the number of patients that would have been screened in the cohort increased threefold to 598, but only 303 (51%) were in fact screened. This drop in percentage is at least partially explained by the fact that most patients were enrolled before the guidelines changed. However, it should be noted that all cases of male breast cancer should have been screened irrespective of guideline version, but less than half of the men in the cohort were referred to screening in the period of this study.

**Fig. 2:**
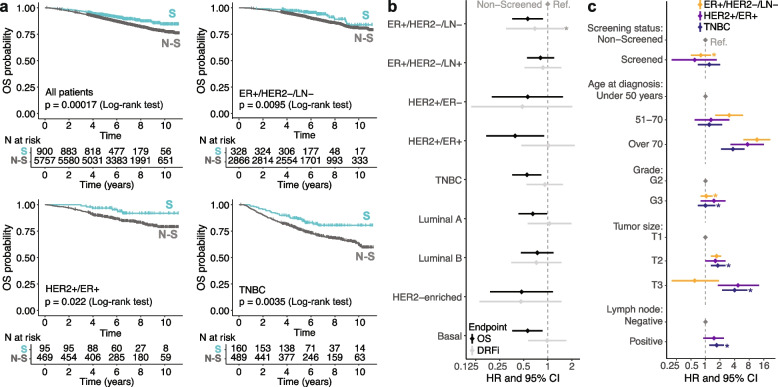
Patient outcome. **a** Kaplan–Meier curves contrasting the two screening subpopulations (S: screened, N-S: non-screened) including all patients in the study and including only patients that belong to the three main clinical subgroups using overall survival as endpoint. **b** Univariate OS and DRFi hazard ratio with 95% confidence interval for screened patients in clinical subgroups/molecular subtypes when using non-screened patients as reference (ref). **c** Multivariate OS hazard ratio with confidence interval for patients in three clinical subgroups. Categories used as reference for each variable are marked in gray. Asterisks in **b** and **c** indicate failure of fulfilling the proportional hazards assumption for Cox regression (*p* < 0.05)

Table 1 outlines the cohort characteristics with respect to clinicopathological variables, ROR scores, and PAM50 molecular subtypes. Based on the complete cohort, all included variables except for tumor size showed statistically significant differences between the two screening subpopulations (corrected chi-square test *p* < 0.001). Screened patients were younger when diagnosed (47.4% were ≤ 50 years versus 16.4%) and enriched for TNBC and PAM50 Basal subtypes compared to non-screened patients. Tumor size did not differ statistically between screening groups, but tumors from screened patients generally had higher NHG status and were more often classified as more proliferative based on the Ki67 biomarker. Screened individuals also presented higher ROR scores. Lastly, screened patients showed better OS than patients that had not been screened for germline variants (log-rank test *p* < 0.001, Fig. 2a), but no difference was observed using DRFi as endpoint (*p* = 0.88, Additional file 3: Fig. S1).

### Can the differences between screening subpopulations be explained by clinically relevant breast cancer subsets?

Breast cancer is a heterogeneous disease that can be further divided into subsets that are associated with specific molecular characteristics. To compare screening subpopulations while accounting for this likely confounder, the SCAN-B cohort was stratified into clinically relevant breast cancer subgroups and PAM50 molecular subtypes totaling nine subsets of patients (Fig. 1, Additional file 2: Table S1). When screened groups within subsets were examined, previously observed differences were less pronounced or not significant (Additional file 2: Table S1). Age at diagnosis was the only variable that differed significantly in all subgroups/subtypes analyzed after multiple testing correction: patients subjected to genetic screening were always younger on average. The only other variable showing significant differences between screening subpopulations was the Ki67 biomarker, for which screened TNBC patients were more often classified as having more proliferative tumors (high Ki67, corrected *p* = 0.01). Within PAM50 molecular subtypes, evidence of a borderline non-significant trend (corrected *p* = 0.05) of higher ROR scores in screened Luminal B patients and higher NHG status in screened Luminal A patients was also observed. To further explore differences between screening subpopulations within relevant clinical and molecular subsets, we analyzed associations with prognosis, gene expression signatures, gene expression pathways, cell type composition, and expressed somatic variants.

#### Prognosis

The OS difference seen in the complete cohort remained significant for ER + /HER2 − /LN − , HER2 + /ER + , TNBC, PAM50 Luminal A, and Basal cases (Fig. 2a, Additional file 3: Fig. S2). Using DRFi as endpoint, survival analyses showed no difference between screening subpopulations for any of the subgroups/subtypes (log-rank *p* > 0.05, Additional file 3: Fig. S1). Univariate Cox regression also showed a significant decrease in the risk of dying in screened patients for those subgroups/subtypes with OS differences (Fig. 2b). To better understand what characteristics may be driving this decrease, multivariate Cox regression was also performed for the three clinical subgroups (Fig. 2c). This revealed significant hazard ratios linked to an increase in the risk of death for older patients and for patients with larger tumors, as well as for TNBC patients whose cancer had spread to lymph nodes, but not related to screening subpopulations.

#### Gene expression signatures

**Fig. 3:**
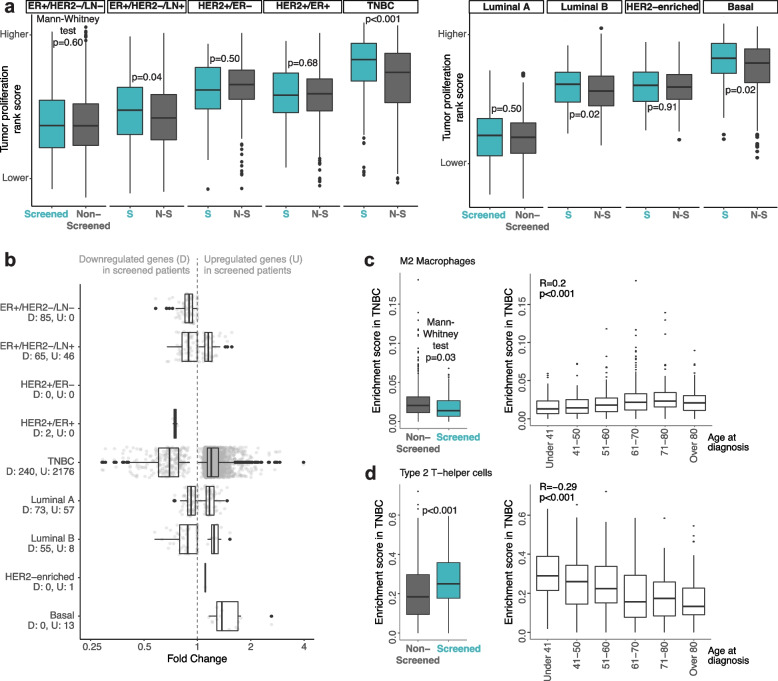
Differences between screening subpopulations within clinical subgroups/PAM50 molecular subtypes found through gene expression data. **a** Distribution of a tumor proliferation measure calculated in silico per sample by screening status of patients and by relevant clinical subgroups or PAM50 molecular subtypes. **b** Fold change distribution and number of differentially expressed genes in screened patients when compared to non-screened patients. **c**, **d** Enrichment scores of two immune cell types by screening status and by age at diagnosis (in years) within all TNBC cases. *R* = Spearman’s correlation coefficient

To verify that screened patients in some breast cancer subsets had more proliferative tumors, we calculated a tumor cell proliferation rank score in silico for each sample (see “Methods”). TNBC cases showed the greatest difference in rank scores between screening subpopulations, with higher proliferation associated with screened patients (Mann–Whitney test *p* < 0.001) corroborating the Ki67 immunohistochemistry results (Fig. 3a). Three other subgroups/subtypes showed moderate evidence of higher tumor proliferation in the screened group, though observed differences were less pronounced.

#### Expression pathways

Supervised gene expression analysis was performed to identify differentially expressed genes (DEGs) between screened and non-screened patients. Eight out of nine patient subsets showed < 130 DEGs between screening subpopulations with an FDR ≤ 0.01, and most identified DEGs showed lower fold change values (Fig. 3b, Additional file 2: Table S3). To further analyze the potential gene expression variance associated with screening status, we performed unsupervised UMAP analysis showing that screening group association does not substantially appear to explain mRNA expression variance in any of the patient subsets (Additional file 3: Fig. S3a), consistent with the differential expression findings. However, in TNBC there were more DEGs between screening groups (Fig. 3b). Gene ontology pathway enrichment analysis of the downregulated genes in TNBC screened patients identified cellular metabolic processes and regulation of these processes as downregulated in the screened group, whereas the analysis of the upregulated genes identified DNA replication, its regulation and double-strand break repair as upregulated in screened TNBC patients (Additional file 2: Table S4).

#### Cell type composition

We investigated whether the tumor microenvironment, and in particular the immune cell landscape, differed between screening subpopulations with two different tools. None of the six immune cell types with proportions estimated by CIBERSORTx differed significantly between screening groups for any breast cancer subgroup/subtype after multiple testing correction (Additional file 2: Table S5). Similarly, no evidence of difference was seen based on the in silico immune response rank score in any patient subset (Additional file 3: Fig. S3b). Using xCell, statistically significant differences after multiple correction were present only in TNBC, where screened patients showed lower enrichment scores of M2 macrophages and higher scores of type 2 T-helper cells (Fig. 3c, d, Additional file 2: Table S5). These differences seem to be driven by the greater number of younger patients in the screened group since scores of both cell types correlated with age (Fig. 3c, d). Of the remaining stromal or epithelial cell types, two cell types not typically associated with breast cancer or breast tissue differed between screening groups: chondrocytes and sebocytes (Additional file 3: Fig. S3c).

#### Expressed somatic variants

**Fig. 4:**
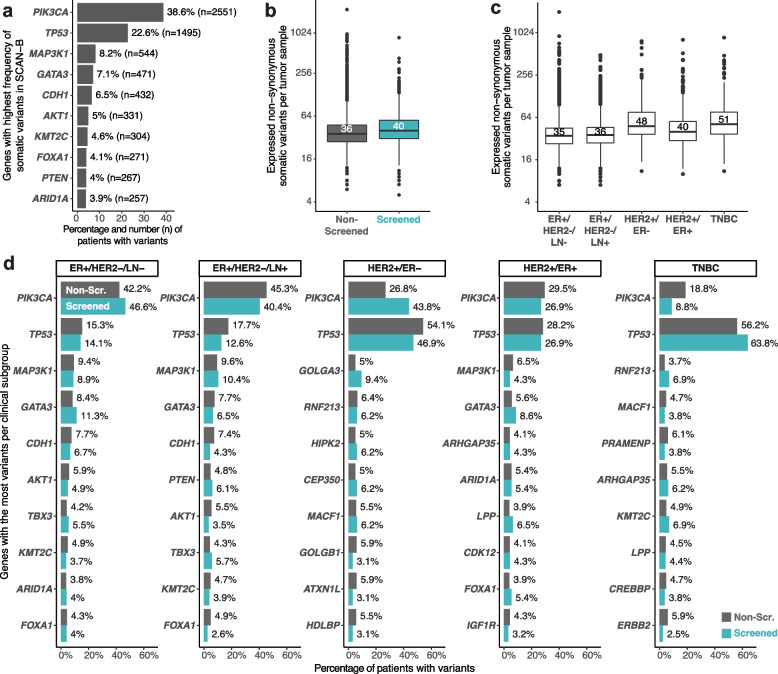
Expressed somatic variants patterns in the cohort. **a** Proportion of SCAN-B patients with expressed somatic variants in the 10 genes with highest number of somatic variants in the cohort. **b**, **c** Distribution of filtered somatic variants found per tumor sample stratified by **b** screening status or by **c** clinical subgroup, with median variant number annotated. **d** Proportion of patients with expressed somatic variants by screening status of patients and by clinical subgroups. Only the 10 genes with the most somatic variants in each subgroup are shown

To analyze differences in the pattern of somatic variants between screening subpopulations, we extracted expressed somatic variants from RNA-seq data for most patients in the cohort (Fig. 4a). Disregarding synonymous variants, the median number of filtered variants found per tumor sample was effectively the same in non-screened and screened subpopulations (Fig. 4b). However, median number of variants varied between clinical subgroups (Fig. 4c). To account for other possible variation in subgroups, we compared frequencies of variants in the 10 genes with most variants in screened and non-screened patients separately for each clinical subgroup (Fig. 4d). The largest difference was observed for *PIK3CA* in TNBC cases, where the screened group showed a lower proportion of patients with somatic variants than the non-screened, a difference that was not significant after multiple testing adjustment (*p* = 0.21) (Additional file 2: Table S6).

In summary, when screening subpopulations were further stratified into the nine subsets, most clinicopathological and prognosis differences observed between screened and non-screened patients at full cohort level were not statistically significant. Further molecular investigations based on RNA-seq data also showed little difference between screening subpopulations in the subsets except for TNBC, where significant differences were found in the gene expression signatures, expression pathways, and immune cell landscape analyses.

### PGVs identified in screened patients and their prevalence within clinical subgroups and molecular subtypes

**Fig. 5:**
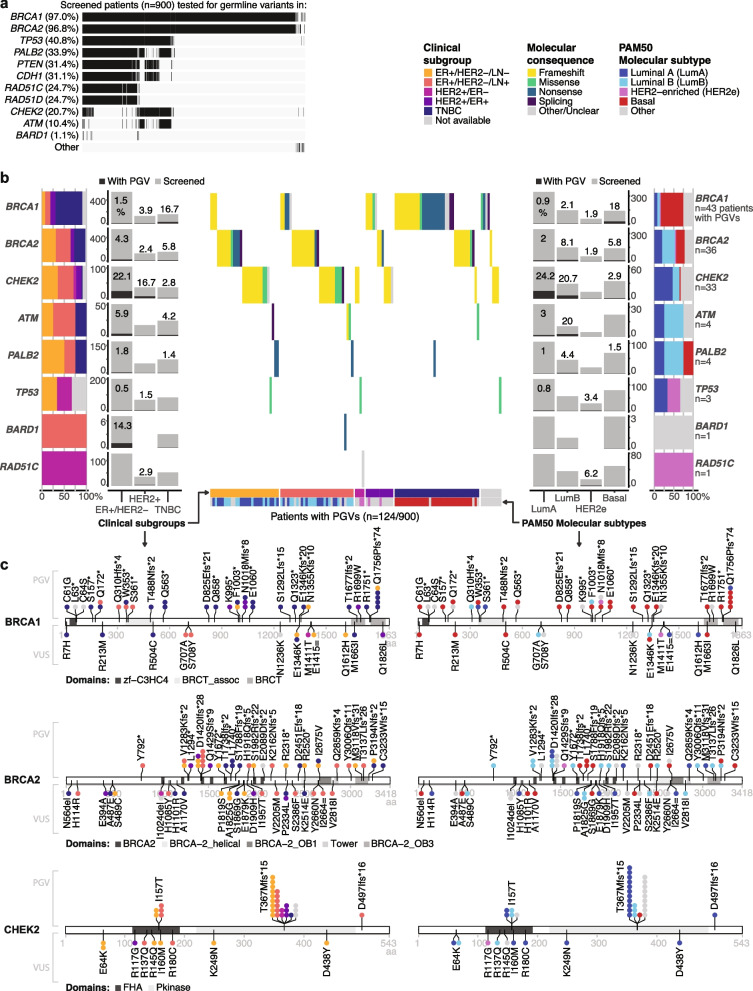
Overview of pathogenic germline variants found in 900 screened patients. **a** Referring clinicians requested different genes be investigated for germline variants in different patients, thus genes were screened at different rates in SCAN-B. **b** Outer horizontal barplots show the distribution of clinical subgroups (left) and molecular subtypes (right) of those with PGVs for each gene of interest that showed at least one patient with PGV in SCAN-B. Vertical barplots show the number of patients with PGVs among those screened specifically for a gene divided by clinical subgroups (left) and molecular subtypes (right). Waterfall plot in the middle shows the predicted molecular consequence of PGVs found in the 124 patients. **c** Lollipop plots showing predicted protein impact of PGVs (above) and variants of uncertain significance (VUS, below) found in *BRCA1*, *BRCA2*, and *CHEK2* colored by clinical subgroup (left) and PAM50 molecular subtype (right). Splicing variants are not shown. Each circle represents one patient with that variant. T367M amino acid modification in *CHEK2* = c.1100delC variant

Results presented above indicate that differences in tested clinicopathological and molecular features between screened and non-screened patients are largely accounted for by the clinical and molecular subsets investigated. However, PGV prevalence is known from literature to differ according to subsets. Therefore, we analyzed the PGV patterns specifically in the screened SCAN-B cohort. Due to current ethical permissions, only variants in genes of interest specific to each patient and reported to referring clinicians were available for matching with clinicopathological and molecular data in this study. These limitations resulted in different screening rates for the 11 genes included here (Fig. 5a, Table 3). In total, 124 screened patients had a PGV detected, and three genes harbored the majority of PGVs in the cohort: *BRCA1*, *BRCA2*, and *CHEK2*—even though the latter was one of the least tested genes (Table 3). The majority of identified PGVs caused frameshift (*n* = 70) or nonsense (*n* = 23) functional changes (Fig. 5b). Figure 5c shows PGVs in *BRCA1*, *BRCA2*, and *CHEK2* including the well-known and here individually most frequent variant *CHEK2* c.1100delC (see Additional file 2: Table S7 for the full variant list).

**Table 3:** PGVs found in patients with full genes screened for germline variants, distributed by clinical subgroup and PAM50 molecular subtype

**Gene**	**Patients** **tested**^a^	**Patients with PGVs within tested**	**ER + /HER2** − **(LN** − **& LN +)**				**HER2 + (ER** − **& ER +)**			**TNBC**			**Not available**
			**All**	**Luminal A**	**Luminal B**	**Other PAM50 subtypes**	**All**	**HER2-enriched**	**Other PAM50 subtypes**	**All**	**Basal**	**Other PAM50 subtypes**	
*BRCA1*	873 (97.0%)	43/873 (4.9%)	8/538 (1.5%)	3/346 (0.9%)	3/146 (2.1%)	2/46 (4.3%)	5/127 (3.9%)	1/52 (1.9%)	4/75 (5.3%)	26/156 (16.7%)	25/139 (18%)	1/17 (5.9%)	4/52 (7.7%)
*BRCA2*	871 (96.8%)	36/871 (4.1%)	23/539 (4.3%)	7/345 (2%)	12/149 (8.1%)	4/45 (8.9%)	3/126 (2.4%)	1/52 (1.9%)	2/74 (2.7%)	9/154 (5.8%)	8/137 (5.8%)	1/17 (5.9%)	1/52 (1.9%)
*TP53*	367 (40.8%)	3/367 (0.8%)	1/202 (0.5%)	1/122 (0.8%)	0/56	0/24	1/67 (1.5%)	1/29 (3.4%)	0/38	0/83	0/79	0/4	1/15 (6.7%)
*PALB2*	305 (33.9%)	4/305 (1.3%)	3/171 (1.8%)	1/105 (1%)	2/45 (4.4%)	0/21	0/51	0/20	0/31	1/73 (1.4%)	1/68 (1.5%)	0/5	0/10
*PTEN*	283 (31.4%)	0/283	0/156	0/91	0/49	0/16	0/51	0/25	0/26	0/63	0/60	0/3	0/13
*CDH1*	280 (31.1%)	0/280	0/154	0/89	0/48	0/17	0/50	0/25	0/25	0/63	0/60	0/3	0/13
*RAD51C*	222 (24.7%)	1/222 (0.5%)	0/128	0/78	0/36	0/14	1/34 (2.9%)	1/16 (6.2%)	0/18	0/52	0/49	0/3	0/8
*RAD51D*	222 (24.7%)	0/222	0/128	0/78	0/36	0/14	0/34	0/16	0/18	0/52	0/49	0/3	0/8
*CHEK2*	186 (20.7%)	33/186 (17.7%)	23/104 (22.1%)	15/62 (24.2%)	6/29 (20.7%)	2/13 (15.4%)	6/36 (16.7%)	0/13	6/23 (26.1%)	1/36 (2.8%)	1/34 (2.9%)	0/2	3/10 (30%)
*ATM*	94 (10.4%)	4/94 (4.3%)	3/51 (5.9%)	1/33 (3%)	2/10 (20%)	0/8	0/17	0/4	0/13	1/24 (4.2%)	0/22	1/2 (50%)	0/2
*BARD1*	10 (1.1%)	1/10 (10.0%)	1/7 (14.3%)	0/3	0/1	1/3 (33.3%)	0/0	0/0	0/0	0/3	0/3	0/0	0/0
All genes	900	124/900 (13.8%)	62/560 (11.1%)	28/359 (7.8%)	25/155 (16.1%)	9/46 (19.6%)	16/128 (12.5%)	4/53 (7.5%)	12/75 (16.0%)	37/160 (23.1%)	34/143 (23.8%)	3/17 (17.6%)	9/52 (17.3%)

^a^ Patients were tested for more genes than reported in this study, but screening results are limited to genes that were of specific interest and reported back to clinicians

PGVs were not equally distributed between clinical subgroups and PAM50 subtypes (Table 3, Fig. 5b, c). Patients with TNBC carried PGVs more often than those with ER + /HER2 − or HER2 + tumors (chi-square *p* = 0.01): on average one PGV every 20 genes tested, nearly twice as often as in ER + /HER2 − (1 in 35) or HER2 + cases (1 in 37). Among the 560 patients with ER + /HER2 − tumors, PGVs were reported more often in patients with PAM50 Luminal B tumors than Luminal A. Moreover, patients with Luminal B cases carried PGVs more often than patients with tumors of other molecular subtypes (chi-square *p* = 0.001) with one PGV in every 24 genes tested. The 128 patients with HER2 + tumors in our cohort presented a percentage of patients with PGVs similar to ER + /HER2 − tumors, few of which belonged to the molecular subtype HER2-enriched. The TNBC subgroup had the highest percentage of patients with PGVs, almost all in the PAM50 Basal subtype.

*BRCA1* PGVs were by far the most frequent germline alteration in screened TNBC patients (Table 3). Within *BRCA1* specifically, PGVs were associated with the TNBC clinical subgroup and the PAM50 Basal subtype (Fisher’s exact tests *p* < 0.001). In our cohort, *BRCA2* PGVs were associated with the Luminal B molecular subtype (Fisher’s exact test *p* = 0.01), but not with any of the clinically relevant subgroups (*p* = 0.53). An association between *CHEK2* PGVs and ER + tumors was seen only when analyzing patients tested specifically for variants in this gene (chi-square test *p* = 0.02). Many variants of uncertain significance for which pathogenicity is still unclear were also detected here (Fig. 5c), and these could alter results if a large number were determined to be pathogenic.

### Do screened patients with PGVs differ from those without PGVs?

**Fig. 6:**
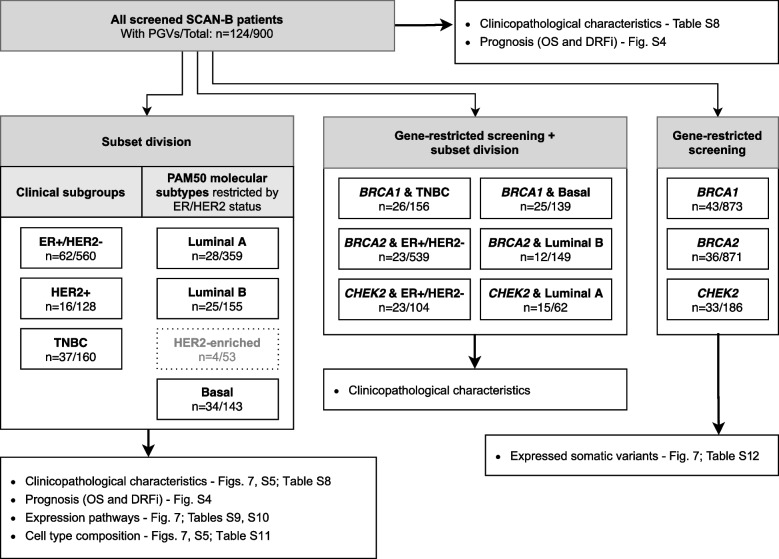
Outline of the comparison within screened patients. Division of screened patients into clinical subgroups and PAM50 molecular subtypes and which information was used to compare those with and without PGVs. Patients without information or belonging to different groups than the ones included are not shown

Based on the availability of matched clinical and molecular data in the SCAN-B cohort, we next analyzed whether clinicopathological and molecular differences existed between screened patients with and without PGVs, using the same methodology as above to compare screening subpopulations. The rationale behind these analyses was that any differences found could potentially be used for improving the screening selection process. Since PGV prevalence varied between clinical and molecular subsets, the investigation was performed at a general level as well as within smaller subsets similar to what was done in the first part of this work (Fig. 6). Considering the entire screened subpopulation, patients carrying PGVs were younger (chi-square corrected *p* = 0.01, five tests) and had tumors with higher Ki67 scores (*p* < 0.001), higher NHG status (grade 3, *p* < 0.001), and higher ROR scores (*p* < 0.001) than patients without PGVs but showed no difference in tumor size (*p* = 0.6) (Additional file 2: Table S8). However, screened patients showed no difference in OS (log-rank *p* = 0.59) nor in DRFi (*p* = 0.27) between those with and without PGVs (Additional file 3: Fig. S4).

**Fig. 7:**
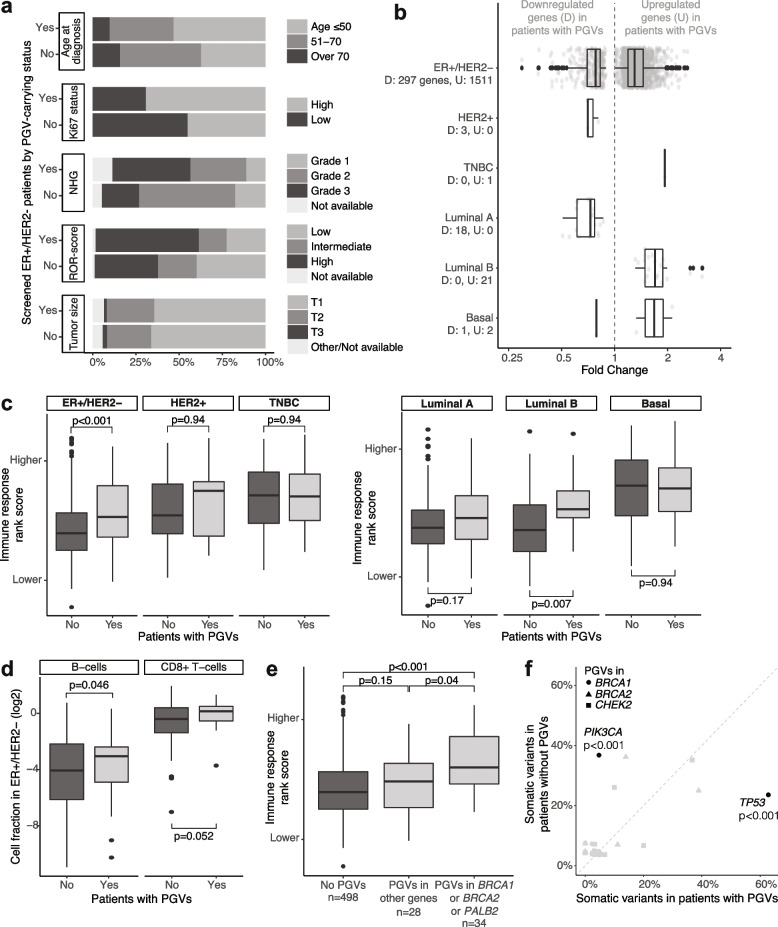
Comparison between screened patients with and without pathogenic germline variants. **a** Distribution of clinicopathological variables in 560 screened patients from the ER + /HER2 − clinical subgroup. **b** Fold change of differentially expressed genes (FDR ≤ 0.01) in patients with PGVs when compared to those without PGVs in clinical subgroups/molecular subtypes that had more than 10 patients with PGVs. **c** Distribution of an immune response measure calculated in silico per sample stratified by patient germline variant status and clinical subgroups/PAM50 molecular subtypes. *p* = corrected Mann–Whitney test p-values, 6 tests. **d** Cell fraction of two immune cell types with statistically significant differences between PGV groups in ER + /HER2 − cases. **e** Distribution of a gene expression immune response score in ER + /HER2 − cases stratified by whether patients had PGVs in specific genes. **f** Proportion of patients with expressed somatic variants in 10 genes stratified by patient germline variant status for *BRCA1*, *BRCA2*, and *CHEK2*. Only genes with a statistically significant difference between groups through Fisher’s exact test are identified

When the comparisons were made within clinical subsets, only PGV-carrying patients with ER + /HER2 − tumors showed the same trends as in the total screening population with higher Ki67 score (chi corrected *p* < 0.01), NHG (*p* = 0.001), ROR score (*p* = 0.01), and in silico proliferation rank scores (Mann–Whitney corrected *p* = 0.001, seven tests) (Fig. 7a, Additional file 2: Table S8, Additional file 3: Fig. S5a). Within PAM50 molecular subtypes with ≥ 10 PGV carriers, a significant difference was only observed in the Luminal B subset, where patients carrying PGVs more often exhibited tumors of higher NHG (grade 3, *p* < 0.01) and higher in silico proliferation (*p* < 0.01) (Additional file 2: Table S8). Finally, there was no difference in OS nor in DRFi between those with and without PGVs when stratified by clinical/molecular subsets (all log-rank *p* > 0.05) (Additional file 3: Fig. S4).

Since different numbers of patients were tested for variants in different genes, we also investigated clinicopathological variables separately for patients that had specific genes tested. This meant that patients were restricted to those screened for germline *BRCA1*, *BRCA2*, or *CHEK2* variants, then split into clinical/molecular subgroups, and then into those with or without PGVs in the tested gene. This resulted in six subsets of interest with ≥ 10 PGV carriers (Fig. 6). Only in *BRCA2*-tested patients with ER + /HER2 − tumors were PGVs associated with higher Ki67 scores (chi-square corrected *p* = 0.02, 30 tests), higher NHG (*p* = 0.01), higher ROR scores (*p* = 0.02), and higher in silico proliferation scores (Mann–Whitney corrected *p* = 0.01, six tests). The last observation regarding tumor proliferation was also seen in patients with and without PGVs for *BRCA2*-tested PAM50 Luminal B cases (Mann–Whitney corrected *p* = 0.01, six tests).

Next, we performed differential gene expression analysis between patients with and without PGVs stratified by clinical subgroups and molecular subtypes. Five subsets showed too few DEGs between groups to be studied further (Fig. 7b, Additional file 2: Table S9). The ER + /HER2 − subgroup, however, had substantially more DEGs that permitted a gene ontology pathway enrichment analysis (Additional file 2: Table S10). In this subgroup, the downregulated genes in cases with PGVs were overrepresented in biological processes such as protein and vesicle localization to the cilium, while the upregulated genes identified, e.g., oxidative phosphorylation, positive regulation of cytokine production, double-strand break repair via break-induced replication, mitotic DNA replication, and positive regulation of T-cell proliferation as upregulated processes.

We also investigated whether the tumor microenvironment differed between patients with and without PGVs within the six clinical/molecular subsets. There was strong evidence of differences in immune composition within ER + /HER2 − and within Luminal B cases based on the in silico immune response rank score for which patients without PGVs showed lower values (Fig. 7c). For specific cell types, differences were found only in the ER + /HER2 − clinical subgroup after multiple testing correction (Additional file 2: Table S11). In this subgroup, the proportion of B-cells differed significantly between PGV groups and the difference in CD8 + T-cells was borderline not significant (Fig. 7d). B-cells were also differentially enriched between PGV groups with xCell, along with eight other cell types such as M1 macrophages and type 2 T-helper cells (Additional file 1: Fig. S5b). While not all statistically significant differences here are expected to be true biological differences (e.g., myocytes were considered different even though their mean/median enrichment scores were < 0.01 in both groups), these analyses indicate that immune response differences could exist between those with and without PGVs. Interestingly, ER + /HER2 − patients with PGVs in genes commonly associated with homologous recombination deficiency such as *BRCA1*,* BRCA2*, and *PALB2* showed higher immune response rank scores than those with PGVs in other genes or without any PGVs in the 11 genes studied (Fig. 7e).

Lastly, we looked for an association between expressed somatic variants and germline variants by comparing patients with and without PGVs in *BRCA1*, *BRCA2*, and *CHEK2* separately without clinical subgroup/molecular subtype restriction (Fig. 7f). This was performed considering all screened patients that had somatic information extracted from RNA-seq (*n* = 893) for the 10 genes with most somatic variants per gene subset. Patients with PGVs in *BRCA1* showed more somatic *TP53* variants and less somatic *PIK3CA* variants than expected when compared to those without any or with benign germline variants (Fisher’s exact test corrected *p* < 0.001). None of the other comparisons performed showed significant association between PGVs and somatic variants after correcting for multiple testing (Additional file 2: Table S12).

In summary, differences between breast cancer patients with and without PGVs in the predisposition genes tested were generally only found in the ER + /HER2 − subgroup and the PAM50 Luminal B subtype. Significant differences included changes in tumor proliferation, immune response scores, immune cell composition, and expression pathways through differentially expressed genes.

## Discussion

In this study, we have comprehensively analyzed the occurrence and distribution of germline alterations in 11 genes associated with higher risk of breast cancer and their impact on the tumor genome through the merging of routine clinical screening data with a transcriptionally profiled population-based breast cancer cohort from a single institution. A particular focus of the current investigation was to analyze whether differences in clinical and molecular features existed between carriers and non-carriers of PGVs, but also between the screened and non-screened subpopulations of patients as partitioned by current screening guidelines.

Controversy exists on how to optimize genetic screening criteria for genes associated with breast cancer risk [7, 10, 11, 46, 47]. Irrespective, current guidelines do not identify all carriers of PGVs, as exemplified by findings in a Swedish population [32, 48]. Risk estimation in women with breast cancer based on features such as tumor markers has been suggested as an important method for identifying screening candidates in the general population [1, 13]. However, for this approach to be fully validated, it appears relevant that the clinicopathological and molecular features of breast tumors in the currently screened and non-screened subpopulations be better understood. A few studies with a population-based approach have studied breast cancer patients with PGVs and further mapped them to detailed clinicopathological variables [13–15, 27]. However, to the best of our knowledge, no large-scale population-based study has been conducted to date that also include high-dimensional genomic data like RNA-seq matched with clinical screening information to allow the investigation of breast cancer transcriptional subtypes, prognostic/predictive gene signatures, differentially expressed genes, expressed somatic variants, and composition of the tumor microenvironment (TME).

It has been reported that breast cancer patients with PGVs have features of more aggressive disease compared to patients with no such alterations [14, 15, 28]. Consistently, the screened subpopulation in our study, likely enriched for patients with PGVs, showed typical features of aggressive disease (e.g., higher NHG, Ki67 status, ROR scores, and specific PAM50 subtypes). These results are expected based on the screening guidelines (e.g., younger patients, diagnosis of TNBC, etc.). However, the screening subpopulations alone are not representative of the full cohort or the breast cancer population from which they were selected. Interestingly, when stratified into relevant clinical subgroups or PAM50 molecular subtypes, differences between screened and non-screened patients in the cohort were mainly non-significant (disregarding that screened patients are generally younger as a clear reflection of screening guidelines) suggesting that stratification variables largely accounted for observed differences. Similarly, our unsupervised and supervised gene expression analyses suggest that screening groups do not represent distinct transcriptional entities when stratified into relevant clinical or molecular subsets, but rather mixes of different biological breast cancer groups. Moreover, we did not find strong evidence of differences in the TME between screening subpopulations in patient subsets. Notably, both for the TME and for the supervised differential gene expression analyses in this study, TNBC was the subset with the clearest differences between screened and non-screened patients. We believe these differences reflect an overrepresentation of a molecular phenotype of TNBC related to DNA repair deficiency (most prominently homologous recombination deficiency, HRD) and characterized by younger disease onset in screened patients compared to a more proposed luminal androgen-like phenotype found more often in older patients (and thus overrepresented in non-screened patients) [49–53].

It has not been fully established whether carriers of PGVs in specific genes are associated with poorer outcome in a general breast cancer context [14, 15, 54, 55]. In our cohort, although screened patients with presumably more PGVs showed a better outcome (OS) than non-screened patients both in general and in some subsets, these differences were not significant when survival analyses were adjusted for variables such as age at diagnosis. Moreover, using DRFi as clinical endpoint, there was no difference between screened and non-screened patients in any subset. Within the screened subpopulation specifically, no difference in patient outcome (OS nor DRFi) was seen between carriers and non-carriers of PGVs in eight genes associated with higher risk of breast cancer. It is important to notice that not finding a PGV in a gene does not mean that the gene is fully functional in a patient’s tumor. In fact, we have recently shown that TNBC cases with somatic *BRCA1* promoter hypermethylation (the main mechanism of HRD in a population-based SCAN-B TNBC cohort [49]) appear as genomic phenocopies of TNBC cases with germline *BRCA1* variants [56]. Notably, in the study by Glodzik et al. [56], we found patients with somatic *BRCA1* promoter hypermethylation more often than carrying inactivating germline *BRCA1* or *BRCA2* variants (35 vs. 26% respectively) in a set of 46 SCAN-B TNBC patients that underwent clinical *BRCA1/BRCA2* genetic screening due to family history and/or young age at diagnosis. Together, these findings could act as confounders in different analyses.

In our cohort, 13.5% (*n* = 900) of patients underwent clinical screening. In a comparative study of unselected breast cancer in Sweden during 2001–2008 by Li et al. [48], 8.2% of 5099 patients were screened as part of clinical practice identifying only 38% of patients with *BRCA1* or *BRCA2* alterations, which had a prevalence of 1.8% in the population. The difference in screening frequency between the two studies is likely due to a specific screening initiative in 2015–2016 in the catchment region of the SCAN-B study [33]. In our cohort, *BRCA1* and *BRCA2* were the most ordered clinical analyses (97% of screened patients) and PGVs were detected in 4.8 and 4.0% of all screened SCAN-B patients (8.8% combined) translating to a prevalence of 0.65 and 0.54% respectively in the entire cohort (1.19% combined). In comparison, when only the clinically tested patients from Li et al. are considered, 8.4% had a *BRCA1*/*BRCA2* alteration corresponding to a prevalence of 0.69% in all 5099 patients [48]. Compared to large population-based studies from other demographic contexts [1, 2], observed PGV frequencies in SCAN-B patients were lower, likely due to incomplete screening of patients. Together, our observed PGV frequencies for *BRCA1*/*BRCA2* appear consistent with a similar demographic context, considering the variations in, e.g., screening referral.

Frequency of PGVs in genes associated with higher risk of tumors has been shown to vary with clinically relevant breast cancer subgroups [13]. For the same 11 genes reported here, Hu et al. [13] reported PGVs in 9.2% of their ~ 55,000 patient cohort distributed in clinical subgroups as follows: 7.6% in ER + /HER2 − (LN + and LN −) cases, 7.3% in HER2 + /ER − , 8.0% in HER2 + /ER + , and 13.2% in TNBC. In SCAN-B, 13.8% of screened patients had PGVs: 11.1% in ER + /HER2 − cases, 12.6% in HER2 + /ER − , 12.1% in HER2 + /ER + , and 23.1% in TNBC. Consistent with Hu et al., TNBC had the highest frequency of PGVs by approximately two-fold. However, our screened cohort is considerably smaller than that of Hu et al. and has a different composition of characteristics such as age at diagnosis and clinical subgroup, which may influence the observed PGV proportions. Had all SCAN-B patients been tested for the genes of interest, percentages reported here would most likely be lower.

This study corroborated reported associations between PGVs in specific genes with clinical (e.g., *CHEK2* and ER + disease) and molecular subtypes (e.g., *BRCA1* and PAM50 Basal) [1, 2, 57]. *BRCA2* PGVs were recently reported to be associated with ER − disease [2], while others have associated these alterations with hormone-driven disease [58–61] including the Luminal B subtype [57], which is consistent with what we found. In our screened cohort, *BRCA2* PGVs were more frequent in ER + /HER2 − Luminal B patients (8.1%) than in TNBC Basal (5.8%) or TNBC patients in general (5.8%), and ER + /HER2 − patients of molecular subtypes other than Luminal A/B had a *BRCA2* PGV frequency of 8.9%, though this was a smaller subset of patients (*n* = 45). Together, this suggests that tumors from patients with *BRCA2* PGVs have more aggressive features. The indication of a higher in silico immune response in ER + /HER2 − tumors with PGVs in prototypical HRD-related genes (*BRCA1*, *BRCA2*, *PALB2*) was also interesting and warrants further investigation in larger cohorts including in situ tissue validation. Moreover, it remains to be validated if this association is due to the high genomic instability associated with HRD in these tumors [22] and whether it has prognostic implications. PGV frequency in the other analyzed genes was too low for association analyses. Also confirming previous studies, PGVs in *BRCA1*/*BRCA2* were distributed across different protein domains of the genes, while the European founder variant c.1100delC was dominating in *CHEK2* [62–64].

The main strengths of this study are the comprehensive available clinical tumor data based on national quality registries, the centralized single institution screening ensuring technical consistency over the years, the population-based cohort with detailed clinicopathological annotation [29, 31, 49], and the depth of molecular profiling based on unbiased mRNA sequencing of fresh tumor tissue. Still, important limitations also apply. The study is limited by germline testing requests made by referring clinicians, i.e., only results from the requested test is accessible for research under current ethical approvals even though a comprehensive multigene NGS panel is used. Thus, we acknowledge that screening rates differ over years depending on guidelines, local initiatives, and SCAN-B study enrollment, and that the study groups do not represent matched case–control groups. Consequently, the true prevalence of PGVs in the SCAN-B catchment region is likely higher than presented here—if all patients had been tested and all NGS panel genes had been reported, results would likely be similar to findings by Li et al. in a similar demographic context [48]. However, it should be noted that while clinical testing results were not available for all genes for all patients, the population frequency of many of these genes is well below 0.75% [2], suggesting that the number of missed PGV carriers would still be very low in our data. As such, the current study represents a population-based view of genetic variants and screening cohort characteristics with real-life limitations from routine healthcare that also include patient and clinician preference. Moreover, despite being the largest study to date connecting high-dimensional transcriptional data to clinical screening, our study is still underpowered for detailed analyses of, e.g., gene-specific PGVs beyond *BRCA1*, *BRCA2*, and *CHEK2*, especially within less common clinical subgroups and PAM50 molecular subtypes. Finally, while all patients in this study had available RNA-seq data, this method is not optimal for deriving a complete view of somatic variants in bulk tumor tissue. A bias in the genes, SNVs, and their frequencies reported herein may be expected compared to DNA-based sequencing, especially concerning alterations causing silencing of tumor suppressor genes that would typically not be detected by variant calling in RNA-seq data.

## Conclusions

The current study has focused on comprehensive high-resolution molecular characterization of a current screening subpopulation (based on real-world clinical decision making) versus non-screened patients using a population-based RNA-seq profiled patient cohort with 6660 individuals, a type of study not reported at this scale to date. We show that while expected clinicopathological differences coupled to screening criteria exist between screening subpopulations, they were mostly non-significant after stratification by clinically relevant breast cancer subgroups and PAM50 molecular subtypes that reflect the known heterogeneity in this tumor type. In addition, we show that there were also no large significant differences between carriers and non-carriers of PGVs when considering relevant subgroups/subtypes. There were, however, some differences in the ER + /HER2 − subgroup when partitioned by PGV status, indicating that there might still be room for improving risk assessment of being a PGV carrier and developing tumors by using other molecular data not currently included in the screening criteria. While this study represents to the best of our knowledge the largest such RNA-seq based study to date, it remains to be determined whether a more focused analysis/profiling using all possible aspects of RNA-seq (e.g., with additional gene signatures, expressed variants, mutational signatures) on a larger cohort of screened patients could identify the molecular traits that would further enrich it for patients with high likelihood of PGVs, thus potentially refining current patient selection through guidelines for screening.

## Supplementary Information

**Additional file 1: Supplementary methods.**

**Additional file 2: Supplementary tables. Table S1.** Clinicopathological characteristics of patients and tumors in SCAN-B divided by screening subpopulation and relevant clinical subgroups/PAM50 molecular subtypes. **Table S2. **Genes included in the original two signatures and whether they were used for calculating the in silico rank scores in our work. **Table S3. **Differentially expressed genes between screening subpopulations by clinical subgroups and PAM50 molecular subtypes. **Table S4. **Significant pathways overrepresented in up- and downregulated genes found in screened TNBC patients compared to non-screened. **Table S5. **Estimated cell fraction and enrichment score mean and standard deviation divided by clinical subgroups and PAM50 molecular subtypes. **Table S6. **Top 10 genes with most expressed somatic variants per clinical subgroup and their distribution in patients with regards to screening status and presence of somatic variants.** Table S7.** List of PGVs and VUS identified in 900 SCAN-B patients screened for germline variants. **Table S8.** Clinicopathological characteristics of patients with and without PGVs.** Table S9.** Differentially expressed genes between patients with and without PGVs divided by clinical subgroups and PAM50 molecular subtypes.** Table S10. **Significant pathways overrepresented in up- and downregulated genes found in ER+/HER2− patients with PGVs when compared to those without variants.** Table S11. **Estimated cell fraction and enrichment score mean and standard deviation by PGV status of patients divided by clinical subgroups and PAM50 molecular subtypes. **Table S12. **Top 10 genes with most expressed somatic variants within patients tested for germline variants in three specific genes and their distribution in patients with regards to presence of somatic variants and PGVs. **Table S13.** All SCAN-B patients used in this study, screening status, for which of the 11 genes analyzed a patient was tested, and whether any PGV was found.

**Additional file 3: Supplementary figures. Figure S1. **Patient outcome in screening subpopulations using distant recurrence-free interval as endpoint. **Figure S2.** Patient outcome in screening subpopulations using overall survival as endpoint. **Figure S3.** Differences found through gene expression data between screened and non-screened patients within subgroups/subtypes. **Figure S4.** Patient outcome in screened patients with and without PGVs. **Figure S5.** Differences found through gene expression data between screened PGV-carriers and non-carriers within clinical subgroups/PAM50 molecular subtypes.

## Data Availability

The datasets supporting the conclusions of this article (clinicopathological, genomic, and transcriptomic data) are available in open repositories as described in original studies [31]. Screening information and presence/absence of pathogenic germline alterations on a patient basis are included in the Additional files of this article (Additional file 2: Table S13), as well as a list of all PGVs and VUS found in the cohort (Additional file 2: Table S7).

## Abbreviations

CI: Confidence interval
DEG: Differentially expressed gene
DRFi: Distant recurrence-free interval
ER: Estrogen receptor
FDR: False discovery rate
FPKM: Fragments per kilobase million
HR: Hazard ratio
HRD: Homologous recombination deficiency
LN: Lymph node
OS: Overall survival
PGV: Pathogenic germline variant
ROR: Risk of recurrence
SNV: Single-nucleotide variant
TME: Tumor microenvironment
TNBC: Triple-negative breast cancer
UMAP: Uniform manifold approximation and projection
VUS: Variant of uncertain significance

## References

[1] Hu C, Hart SN, Gnanaolivu R, Huang H, Lee KY, Na J (2021). A population-based study of genes previously implicated in breast cancer. N Engl J Med.

[2] Dorling L, Carvalho S, Allen J, Gonzalez-Neira A, Luccarini C, Breast Cancer Association C (2021). Breast cancer risk genes - association analysis in more than 113,000 women. N Engl J Med.

[3] Niravath P, Cakar B, Ellis M (2017). The role of genetic testing in the selection of therapy for breast cancer: a review. JAMA Oncol.

[4] Domchek SM, Friebel TM, Singer CF, Evans DG, Lynch HT, Isaacs C (2010). Association of risk-reducing surgery in BRCA1 or BRCA2 mutation carriers with cancer risk and mortality. JAMA.

[5] Tung NM, Garber JE (2018). BRCA1/2 testing: therapeutic implications for breast cancer management. Br J Cancer.

[6] Owens DK, Davidson KW, Krist AH, Barry MJ, Cabana M, Force USPST (2019). Risk assessment, genetic counseling, and genetic testing for BRCA-related cancer: US Preventive Services Task Force Recommendation Statement. JAMA.

[7] Daly MB, Pal T, Berry MP, Buys SS, Dickson P, Domchek SM (2021). Genetic/familial high-risk assessment: breast, ovarian, and pancreatic, Version 2.2021, NCCN Clinical Practice Guidelines in Oncology. J Natl Compr Canc Netw.

[8] King MC, Levy-Lahad E, Lahad A (2014). Population-based screening for BRCA1 and BRCA2: 2014 Lasker Award. JAMA.

[9] Gabai-Kapara E, Lahad A, Kaufman B, Friedman E, Segev S, Renbaum P (2014). Population-based screening for breast and ovarian cancer risk due to BRCA1 and BRCA2. Proc Natl Acad Sci U S A.

[10] Manahan ER, Kuerer HM, Sebastian M, Hughes KS, Boughey JC, Euhus DM (2019). Consensus guidelines on genetic testing for hereditary breast cancer from the American Society of Breast Surgeons. Ann Surg Oncol.

[11] Daly MB, Pilarski R, Yurgelun MB, Berry MP, Buys SS, Dickson P (2020). NCCN Guidelines Insights: genetic/familial high-risk assessment: breast, ovarian, and pancreatic, Version 1.2020. J Natl Compr Canc Netw.

[12] Parker JS, Mullins M, Cheang MC, Leung S, Voduc D, Vickery T (2009). Supervised risk predictor of breast cancer based on intrinsic subtypes. J Clin Oncol.

[13] Hu C, Polley EC, Yadav S, Lilyquist J, Shimelis H, Na J (2020). The contribution of germline predisposition gene mutations to clinical subtypes of invasive breast cancer from a clinical genetic testing cohort. J Natl Cancer Inst.

[14] Ho PJ, Khng AJ, Loh HW, Ho WK, Yip CH, Mohd-Taib NA (2021). Germline breast cancer susceptibility genes, tumor characteristics, and survival. Genome Med.

[15] Li J, Ugalde-Morales E, Wen WX, Decker B, Eriksson M, Torstensson A (2018). Differential burden of rare and common variants on tumor characteristics, survival, and mode of detection in breast cancer. Cancer Res.

[16] de Bock GH, Mourits MJ, Schutte M, Krol-Warmerdam EM, Seynaeve C, Blom J (2006). Association between the CHEK2*1100delC germ line mutation and estrogen receptor status. Int J Gynecol Cancer.

[17] Domagala P, Wokolorczyk D, Cybulski C, Huzarski T, Lubinski J, Domagala W (2012). Different CHEK2 germline mutations are associated with distinct immunophenotypic molecular subtypes of breast cancer. Breast Cancer Res Treat.

[18] Shimelis H, LaDuca H, Hu C, Hart SN, Na J, Thomas A (2018). Triple-negative breast cancer risk genes identified by multigene hereditary cancer panel testing. J Natl Cancer Inst.

[19] Couch FJ, Hart SN, Sharma P, Toland AE, Wang X, Miron P (2015). Inherited mutations in 17 breast cancer susceptibility genes among a large triple-negative breast cancer cohort unselected for family history of breast cancer. J Clin Oncol.

[20] Buys SS, Sandbach JF, Gammon A, Patel G, Kidd J, Brown KL (2017). A study of over 35,000 women with breast cancer tested with a 25-gene panel of hereditary cancer genes. Cancer.

[21] Nik-Zainal S, Alexandrov LB, Wedge DC, Van Loo P, Greenman CD, Raine K (2012). Mutational processes molding the genomes of 21 breast cancers. Cell.

[22] Nik-Zainal S, Davies H, Staaf J, Ramakrishna M, Glodzik D, Zou X (2016). Landscape of somatic mutations in 560 breast cancer whole-genome sequences. Nature.

[23] Southey MC, Dowty JG, Riaz M, Steen JA, Renault AL, Tucker K (2021). Population-based estimates of breast cancer risk for carriers of pathogenic variants identified by gene-panel testing. NPJ Breast Cancer.

[24] Momozawa Y, Iwasaki Y, Parsons MT, Kamatani Y, Takahashi A, Tamura C (2018). Germline pathogenic variants of 11 breast cancer genes in 7,051 Japanese patients and 11,241 controls. Nat Commun.

[25] Tung N, Lin NU, Kidd J, Allen BA, Singh N, Wenstrup RJ (2016). Frequency of germline mutations in 25 cancer susceptibility genes in a sequential series of patients with breast cancer. J Clin Oncol.

[26] Kurian AW, Ward KC, Howlader N, Deapen D, Hamilton AS, Mariotto A (2019). Genetic testing and results in a population-based cohort of breast cancer patients and ovarian cancer patients. J Clin Oncol.

[27] Hauke J, Horvath J, Gross E, Gehrig A, Honisch E, Hackmann K (2018). Gene panel testing of 5589 BRCA1/2-negative index patients with breast cancer in a routine diagnostic setting: results of the German Consortium for Hereditary Breast and Ovarian Cancer. Cancer Med.

[28] Mavaddat N, Dorling L, Carvalho S, Allen J, Gonzalez-Neira A, Breast Cancer Association C (2022). Pathology of tumors associated with pathogenic germline variants in 9 breast cancer susceptibility genes. JAMA Oncol.

[29] Ryden L, Loman N, Larsson C, Hegardt C, Vallon-Christersson J, Malmberg M (2018). Minimizing inequality in access to precision medicine in breast cancer by real-time population-based molecular analysis in the SCAN-B initiative. Br J Surg.

[30] Saal LH, Vallon-Christersson J, Hakkinen J, Hegardt C, Grabau D, Winter C (2015). The Sweden Cancerome Analysis Network - Breast (SCAN-B) Initiative: a large-scale multicenter infrastructure towards implementation of breast cancer genomic analyses in the clinical routine. Genome Med.

[31] Staaf J, Hakkinen J, Hegardt C, Saal LH, Kimbung S, Hedenfalk I (2022). RNA sequencing-based single sample predictors of molecular subtype and risk of recurrence for clinical assessment of early-stage breast cancer. NPJ Breast Cancer.

[32] Nilsson MP, Winter C, Kristoffersson U, Rehn M, Larsson C, Saal LH (2017). Efficacy versus effectiveness of clinical genetic testing criteria for BRCA1 and BRCA2 hereditary mutations in incident breast cancer. Fam Cancer.

[33] Nilsson MP, Torngren T, Henriksson K, Kristoffersson U, Kvist A, Silfverberg B (2018). BRCAsearch: written pre-test information and BRCA1/2 germline mutation testing in unselected patients with newly diagnosed breast cancer. Breast Cancer Res Treat.

[34] ClinVar. Available from: https://www.ncbi.nlm.nih.gov/clinvar/.

[35] Gu Z, Eils R, Schlesner M (2016). Complex heatmaps reveal patterns and correlations in multidimensional genomic data. Bioinformatics.

[36] Ou J, Zhu LJ (2019). trackViewer: a Bioconductor package for interactive and integrative visualization of multi-omics data. Nat Methods.

[37] Mistry J, Chuguransky S, Williams L, Qureshi M, Salazar GA, Sonnhammer ELL (2021). Pfam: The protein families database in 2021. Nucleic Acids Res.

[38] Carter SL, Eklund AC, Kohane IS, Harris LN, Szallasi Z (2006). A signature of chromosomal instability inferred from gene expression profiles predicts clinical outcome in multiple human cancers. Nat Genet.

[39] Nacer DF, Liljedahl H, Karlsson A, Lindgren D, Staaf J. Pan-cancer application of a lung-adenocarcinoma-derived gene-expression-based prognostic predictor. Brief Bioinform. 2021;22(6):bbab154. 10.1093/bib/bbab154 PMC8574611 33971670

[40] Fredlund E, Staaf J, Rantala JK, Kallioniemi O, Borg A, Ringner M (2012). The gene expression landscape of breast cancer is shaped by tumor protein p53 status and epithelial-mesenchymal transition. Breast Cancer Res.

[41] Tusher VG, Tibshirani R, Chu G (2001). Significance analysis of microarrays applied to the ionizing radiation response. Proc Natl Acad Sci U S A.

[42] PANTHER Classification System. Available from: http://pantherdb.org.

[43] Aran D, Hu Z, Butte AJ (2017). xCell: digitally portraying the tissue cellular heterogeneity landscape. Genome Biol.

[44] Newman AM, Steen CB, Liu CL, Gentles AJ, Chaudhuri AA, Scherer F (2019). Determining cell type abundance and expression from bulk tissues with digital cytometry. Nat Biotechnol.

[45] Brueffer C, Gladchuk S, Winter C, Vallon-Christersson J, Hegardt C, Hakkinen J (2020). The mutational landscape of the SCAN-B real-world primary breast cancer transcriptome. EMBO Mol Med.

[46] Yadav S, Hu C, Hart SN, Boddicker N, Polley EC, Na J (2020). Evaluation of germline genetic testing criteria in a hospital-based series of women with breast cancer. J Clin Oncol.

[47] Familial breast cancer: classification, care and managing breast cancer and related risks in people with a family history of breast cancer. London: National Institute for Health and Care Excellence (NICE): Guidelines. 2019. 31940157

[48] Li J, Wen WX, Eklund M, Kvist A, Eriksson M, Christensen HN (2019). Prevalence of BRCA1 and BRCA2 pathogenic variants in a large, unselected breast cancer cohort. Int J Cancer.

[49] Staaf J, Glodzik D, Bosch A, Vallon-Christersson J, Reutersward C, Hakkinen J (2019). Whole-genome sequencing of triple-negative breast cancers in a population-based clinical study. Nat Med.

[50] Aine M, Boyaci C, Hartman J, Hakkinen J, Mitra S, Campos AB (2021). Molecular analyses of triple-negative breast cancer in the young and elderly. Breast Cancer Res.

[51] Lehmann BD, Bauer JA, Chen X, Sanders ME, Chakravarthy AB, Shyr Y (2011). Identification of human triple-negative breast cancer subtypes and preclinical models for selection of targeted therapies. J Clin Invest.

[52] Bareche Y, Buisseret L, Gruosso T, Girard E, Venet D, Dupont F (2020). Unraveling triple-negative breast cancer tumor microenvironment heterogeneity: towards an optimized treatment approach. J Natl Cancer Inst.

[53] Bareche Y, Venet D, Ignatiadis M, Aftimos P, Piccart M, Rothe F (2018). Unravelling triple-negative breast cancer molecular heterogeneity using an integrative multiomic analysis. Ann Oncol.

[54] Baretta Z, Mocellin S, Goldin E, Olopade OI, Huo D (2016). Effect of BRCA germline mutations on breast cancer prognosis: a systematic review and meta-analysis. Medicine (Baltimore).

[55] Wang YA, Jian JW, Hung CF, Peng HP, Yang CF, Cheng HS (2018). Germline breast cancer susceptibility gene mutations and breast cancer outcomes. BMC Cancer.

[56] Glodzik D, Bosch A, Hartman J, Aine M, Vallon-Christersson J, Reutersward C (2020). Comprehensive molecular comparison of BRCA1 hypermethylated and BRCA1 mutated triple negative breast cancers. Nat Commun.

[57] Jonsson G, Staaf J, Vallon-Christersson J, Ringner M, Gruvberger-Saal SK, Saal LH (2012). The retinoblastoma gene undergoes rearrangements in BRCA1-deficient basal-like breast cancer. Cancer Res.

[58] Mavaddat N, Barrowdale D, Andrulis IL, Domchek SM, Eccles D, Nevanlinna H (2012). Pathology of breast and ovarian cancers among BRCA1 and BRCA2 mutation carriers: results from the Consortium of Investigators of Modifiers of BRCA1/2 (CIMBA). Cancer Epidemiol Biomarkers Prev.

[59] Lakhani SR, Jacquemier J, Sloane JP, Gusterson BA, Anderson TJ, van de Vijver MJ (1998). Multifactorial analysis of differences between sporadic breast cancers and cancers involving BRCA1 and BRCA2 mutations. J Natl Cancer Inst.

[60] Lakhani SR, Reis-Filho JS, Fulford L, Penault-Llorca F, van der Vijver M, Parry S (2005). Prediction of BRCA1 status in patients with breast cancer using estrogen receptor and basal phenotype. Clin Cancer Res.

[61] Chen B, Zhang G, Li X, Ren C, Wang Y, Li K (2020). Comparison of BRCA versus non-BRCA germline mutations and associated somatic mutation profiles in patients with unselected breast cancer. Aging (Albany NY).

[62] Stolarova L, Kleiblova P, Janatova M, Soukupova J, Zemankova P, Macurek L, et al. CHEK2 germline variants in cancer predisposition: stalemate rather than checkmate. Cells. 2020;9(12):2675. 10.3390/cells9122675 PMC7763663 33322746

[63] Nurmi A, Muranen TA, Pelttari LM, Kiiski JI, Heikkinen T, Lehto S (2019). Recurrent moderate-risk mutations in Finnish breast and ovarian cancer patients. Int J Cancer.

[64] Sutcliffe EG, Stettner AR, Miller SA, Solomon SR, Marshall ML, Roberts ME (2020). Differences in cancer prevalence among CHEK2 carriers identified via multi-gene panel testing. Cancer Genet.

