# Supplementary material for: Molecular characteristics of breast tumors in patients screened for germline predisposition from a population-based observational study
Source: Genome Med. 2023 Apr 14;15:25. doi: 10.1186/s13073-023-01177-4 (PMC10103478; doi:10.1186/s13073-023-01177-4)
Supplement: Supplementary file 1 — Additional file 1: Supplementary methods. [file 13073_2023_1177_MOESM1_ESM.pdf]

Supplementary Methods

Unselected population-based breast cancer cohort

Specific patient inclusion and exclusion criteria for the SCAN-B cohort are reported in the original publication[1]. For this work, patients were divided into clinically relevant subgroups based on ER, PR, and HER2 status (+ = positive, - = negative). Whether cancer has spread to lymph nodes (LN) influences treatment choice for patients with ER+/HER2- disease. Based on the size of the SCAN-B cohort, patients with ER+/HER2- disease could therefore be further divided based on LN status without loss of statistical power, unlike those with other clinical subgroups. This resulted in a final stratification of the cohort into the five subgroups mentioned in the article: (i) ER+/HER2-/LN-, (ii) ER+/HER2-/LN+, (iii) HER2+/ER-, (iv) HER2+/ER+, and (v) TNBC. Four percent of patients had missing data on one or more of the ER/HER2/LN variables excluding them from analyses focused on clinical subgroups. For analyses conducted within germline screened patients alone, the five clinical subgroups were combined into only three for larger sample size: (i) ER+/HER2-, including both LN- and LN+; (ii) HER2+, including both ER- and ER+; and (iii) TNBC. Finally, the PAM50 classification was constrained by ER/HER2 status, i.e., the PAM50 subtypes used in this work refer to tumors that are: (i) Luminal A and ER+/HER2-, (ii) Luminal B and ER+/HER2-, (iii) HER2-enriched and HER2+, (iv) Basal and TNBC. Consequently, 1198 (18%) patients were excluded from analyses using PAM50 molecular subtypes due to lack of ER/HER2 information or for having other receptor/subtype combinations.

Screening for variants in predisposition genes

Screening was performed during or after diagnosis for most patients (Fig. SM1a). Most screened patients (n=863/900) were analyzed for PGVs in several genes through NGS-based

hybrid capture panels[2] that included the 11 genes cited in the manuscript. However, some patients were tested only for a single variant found in a family member (n=28) or only for a set of founder variants (n=2). Remaining patients had first been screened at other institutions (n=7) with a single variant later confirmed by the laboratory unit at the Division of Oncology. On average, between 6.1 and 10.5% of patients enrolled in SCAN-B in a calendar year were screened, except during the biennium 2015-2016, when over 20% of patients were screened due to a specific regional initiative[2] (Fig. SM1b). No significant difference in the proportion of patients screened was observed after the 2017 guideline revision.

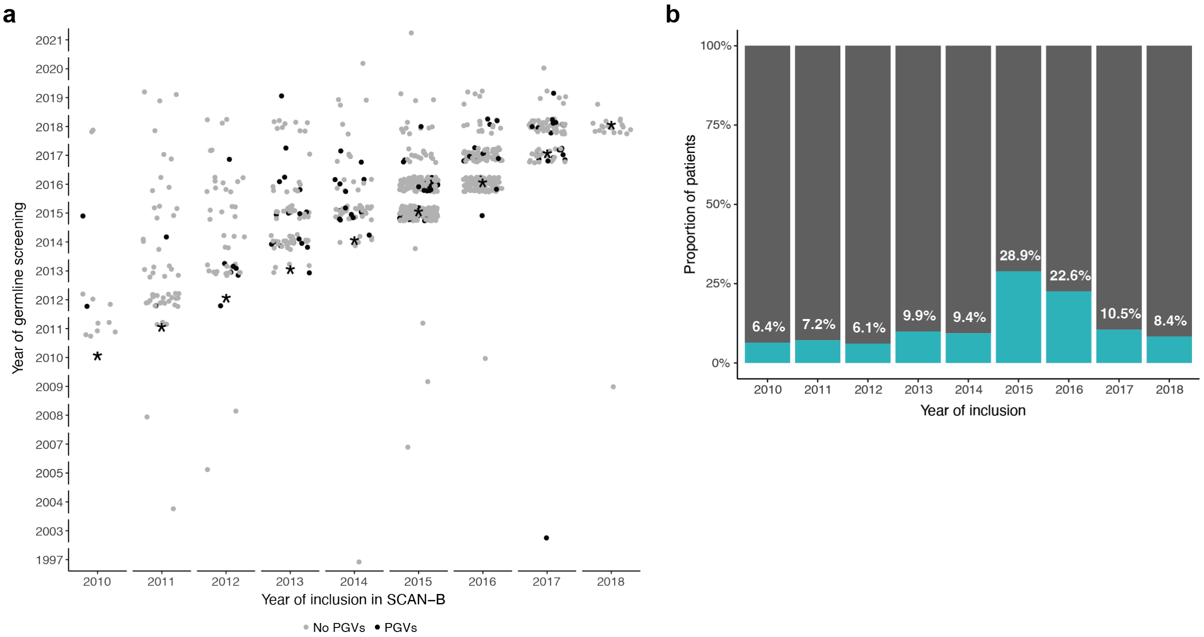

Figure SM1. Screening. (a) Most of the 861 patients with screening time point were screened during or after breast cancer diagnosis. Asterisks mark same year in the two axes. (b) Screened subpopulation proportion per year in SCAN-B.

Gene expression analyses

Processed RNA-seq data (fragments per kilobase million, FPKM) were used to classify 1,861 samples without information for the Ki67 tumor cell proliferation biomarker into having Low or High proliferation by comparing *MKI67* expression values to those of samples already

classified by immunohistochemistry staining from Staaf et al.[1] This was done by plotting the distribution of expression values for both categories and setting a threshold between them (Fig. SM2a). To calculate another tumor proliferation measure, expression values of genes belonging to the CIN70 signature[3] (Supplementary Table S2, Additional File 2) were used to estimate rank scores per sample. Briefly, 19,292 genes were ranked from lower to higher expression according to their FPKM values independently for each sample and the ranks of 72 approved gene symbols derived from the original signature (Supplementary Table S2, Additional File 2) were added together to create a per sample rank score. This proliferation rank score was higher in samples with higher Nottingham Histological Grade (NHG) and higher Ki67 status (Fig. SM2b), allowing the use of this scoring system within SCAN-B samples. The same approach but using other genes (Supplementary Table S2, Additional File 2) was used to calculate the *in silico* immune response rank score.

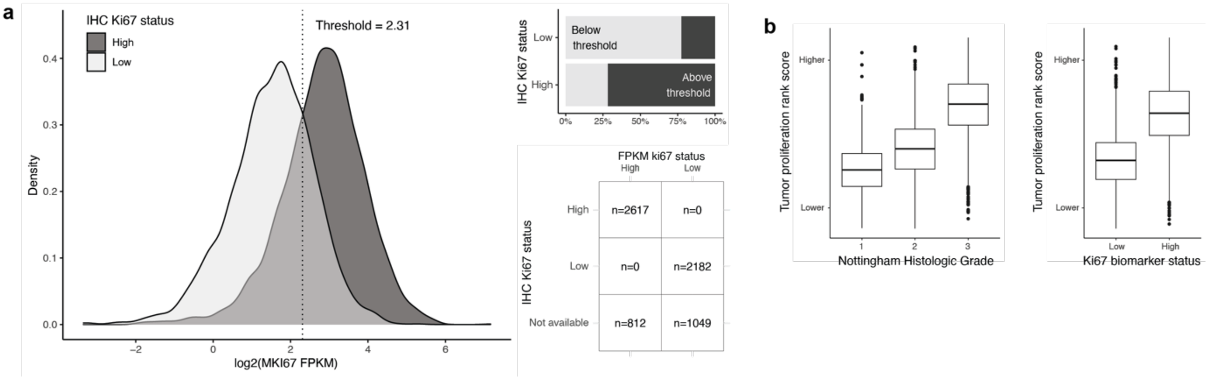

Figure SM2. Proliferation markers. (a) Threshold between *MKI67* FPKM values of samples classified as Low and High Ki67 status according to immunohistochemistry information. (b) Dispersion of a tumor proliferation measure calculated *in silico* per sample in different tumor grade and Ki67 biomarker categories.

Differential gene expression analyses between screening groups were performed as follows: for each breast cancer subset, FPKM values from the samples included

were (i) offset by 0.1, (ii) log2-transformed, (iii) and median-centered per each of the 19,675 Ensembl identifiers. Delta values were chosen so that the FDR would be as equal to 0.01 as possible. To identify pathways based on up- or down-regulated genes, we used the GO-Slim Biological Process as annotation data set, *Homo sapiens* genes as reference list, Fisher's exact test and FDR correction. Gene ontology terms with FDR $p<0.05$ were considered significant. Finally, for the UMAP analysis, we first performed feature selection by keeping only genes with an FPKM of at least 1 in at least 95% of samples, which left an average of ~14,809 genes per subset. We then proceeded with the recipe including a normalization step ("step_normalize()") and the UMAP step ("step_umap()"), both including all predictors/genes.

When estimating cell types per sample, all 6,660 samples were quantified at once to avoid biases. CIBERSORTx results were filtered to keep only samples with $p<0.05$ (n=4591 kept samples) since lower p-values are connected to a more reliable deconvolution process. Cell quantification between screening subpopulations was compared with the Mann-Whitney test for each cell type and then corrected for multiple testing with the Bonferroni method. The correction was performed for all clinical subgroups together (once for each method) and for all PAM50 molecular subtypes together (once for each method). P-values in all other analyses were corrected whenever necessary using the less strict Benjamini-Hochberg method. To calculate the correlation between cell proportions obtained with xCell and age at diagnosis, the Spearman correlation test was used with ages rounded up to 5-year installments (e.g., 31-35 = 35, 36-40 = 40).

Lastly, RNA-seq data were also used to identify expressed somatic variants in 6,614 patients. The pipeline used varied from what was reported in Brueffer et al.[4] through the following parameters. Firstly, a different alignment strategy was used (a pre-filtering masking step was included and the *-data* parameter was excluded yielding less raw variants).

Secondly, databases used for variant annotation were updated to dbSNP[5] v153, COSMIC[6] v90, and gnomAd[7] v2.1.1, and the panel of non-tumoral breast tissues was expanded to include 26 samples instead of the original 10. Finally, minor changes were also made to the variant filtering step: the G5 flag was removed in the current dbSNP version and is therefore no longer part of the filtering expression; and expressions involving the SCAN-B database were modified to rely on frequencies instead of counts (e.g., CNT_NR <= 3 was changed to scanb_MF_NR <= 0.001). As part of the pipeline, somatic variants were annotated with SnpEff[8], a software that returns among other information which genes are affected by the variant and predicted functional impacts such as synonymous amino acid changes, missense, nonsense, frameshift, affecting untranslated regions, etc. Whenever a variant had more than one effect predicted, the most deleterious effect (appearing at the top of the list) with the most information available (e.g., amino acid change) was selected to be used in the analyses.

Statistical methods

Boxplot elements correspond to: (i) center line = median, (ii) box limits = upper and lower quartiles, (iii) whiskers = 1.5x interquartile range.

References

1. Staaf J, Hakkinen J, Hegardt C, Saal LH, Kimbung S, Hedenfalk I, et al. RNA sequencing-based single sample predictors of molecular subtype and risk of recurrence for clinical assessment of early-stage breast cancer. NPJ Breast Cancer. 2022;8(1):94.

2. Nilsson MP, Torngren T, Henriksson K, Kristoffersson U, Kvist A, Silfverberg B, et al. BRCAsearch: written pre-test information and BRCA1/2 germline mutation testing in unselected patients with newly diagnosed breast cancer. Breast Cancer Res Treat. 2018;168(1):117-26.

3. Carter SL, Eklund AC, Kohane IS, Harris LN, Szallasi Z. A signature of chromosomal instability inferred from gene expression profiles predicts clinical outcome in multiple human cancers. Nat Genet. 2006;38(9):1043-8.

4. Brueffer C, Gladchuk S, Winter C, Vallon-Christersson J, Hegardt C, Hakkinen J, et al. The mutational landscape of the SCAN-B real-world primary breast cancer transcriptome. EMBO Mol Med. 2020;12(10):e12118.

5. Sherry ST, Ward MH, Kholodov M, Baker J, Phan L, Smigielski EM, et al. dbSNP: the NCBI database of genetic variation. Nucleic Acids Res. 2001;29(1):308-11.

6. Tate JG, Bamford S, Jubb HC, Sondka Z, Beare DM, Bindal N, et al. COSMIC: the Catalogue Of Somatic Mutations In Cancer. Nucleic Acids Res. 2019;47(D1):D941-D7.

7. Collins RL, Brand H, Karczewski KJ, Zhao X, Alfoldi J, Francioli LC, et al. A structural variation reference for medical and population genetics. Nature. 2020;581(7809):444-51.

8. Cingolani P, Platts A, Wang le L, Coon M, Nguyen T, Wang L, et al. A program for annotating and predicting the effects of single nucleotide polymorphisms, SnpEff: SNPs in the genome of Drosophila melanogaster strain w1118; iso-2; iso-3. Fly (Austin). 2012;6(2):80-92.
