# Supplementary material for: Molecular characteristics of breast tumors in patients screened for germline predisposition from a population-based observational study
Source: Genome Med. 2023 Apr 14;15:25. doi: 10.1186/s13073-023-01177-4 (PMC10103478; doi:10.1186/s13073-023-01177-4)
Supplement: Supplementary file 3 — Additional file 3: Supplementary figures. Figure S1. Patient outcome in screening subpopulations using distant recurrence-free interval as endpoint. Figure S2. Patient outcome in screening subpopulations using overall survival as endpoint. Figure S3. Differences found through gene expression data between screened and non-screened patients within subgroups/subtypes. Figure S4. Patient outcome in screened patients with and without PGVs. Figure S5. Differences found through gene expression data between screened PGV-carriers and non-carriers within clinical subgroups/PAM50 molecular subtypes. [file 13073_2023_1177_MOESM3_ESM.pdf]

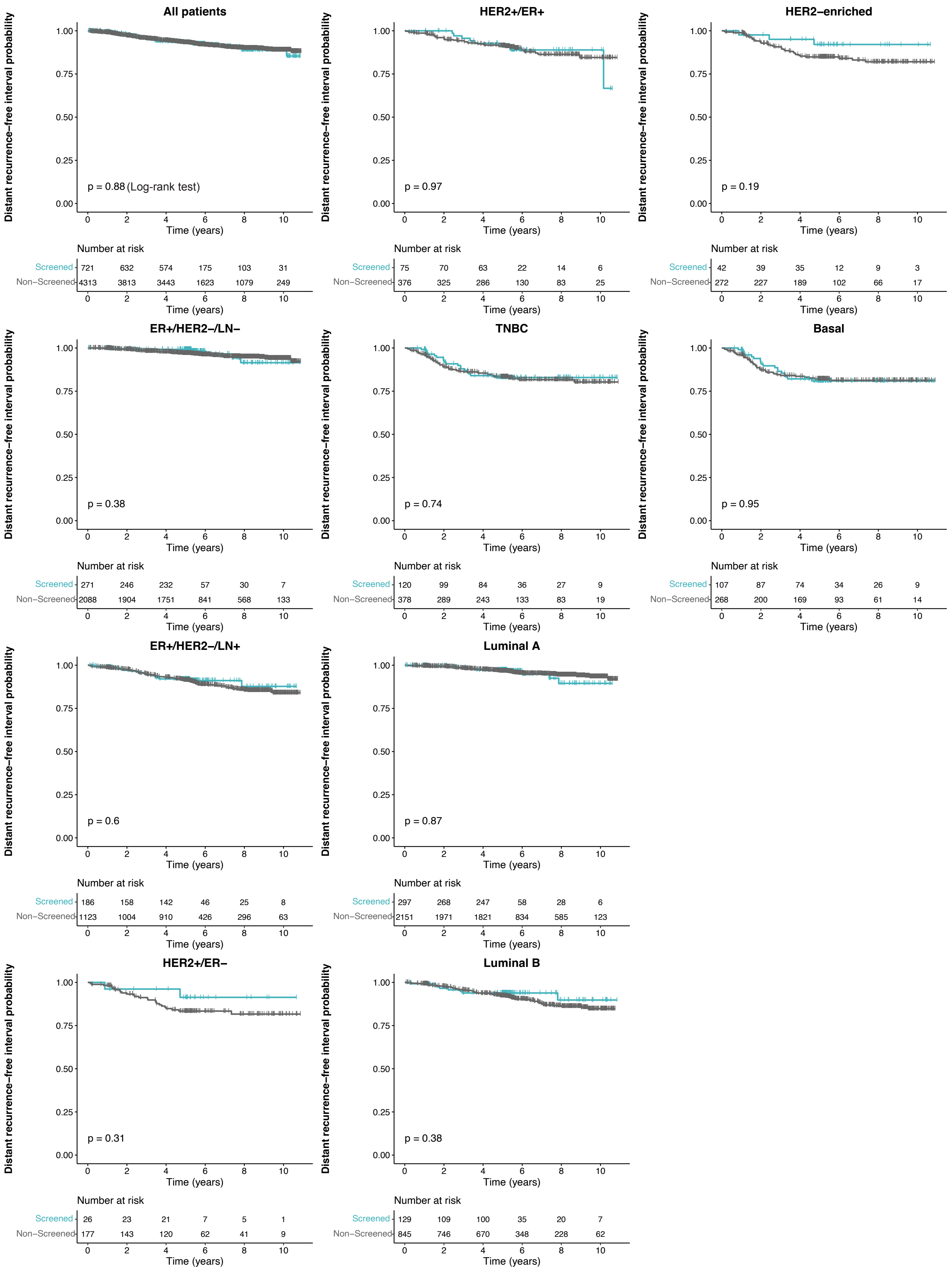

Figure S1. Patient outcome in screening subpopulations using a different endpoint. Kaplan-Meier curves using distant recurrence-free interval as endpoint contrasting patients that have and have not been screened for germline variants in 11 genes associated with higher risk of breast cancer. Curves include all patients in the study, patients divided in five relevant breast cancer clinical subgroups, and in four PAM50 molecular subtypes.

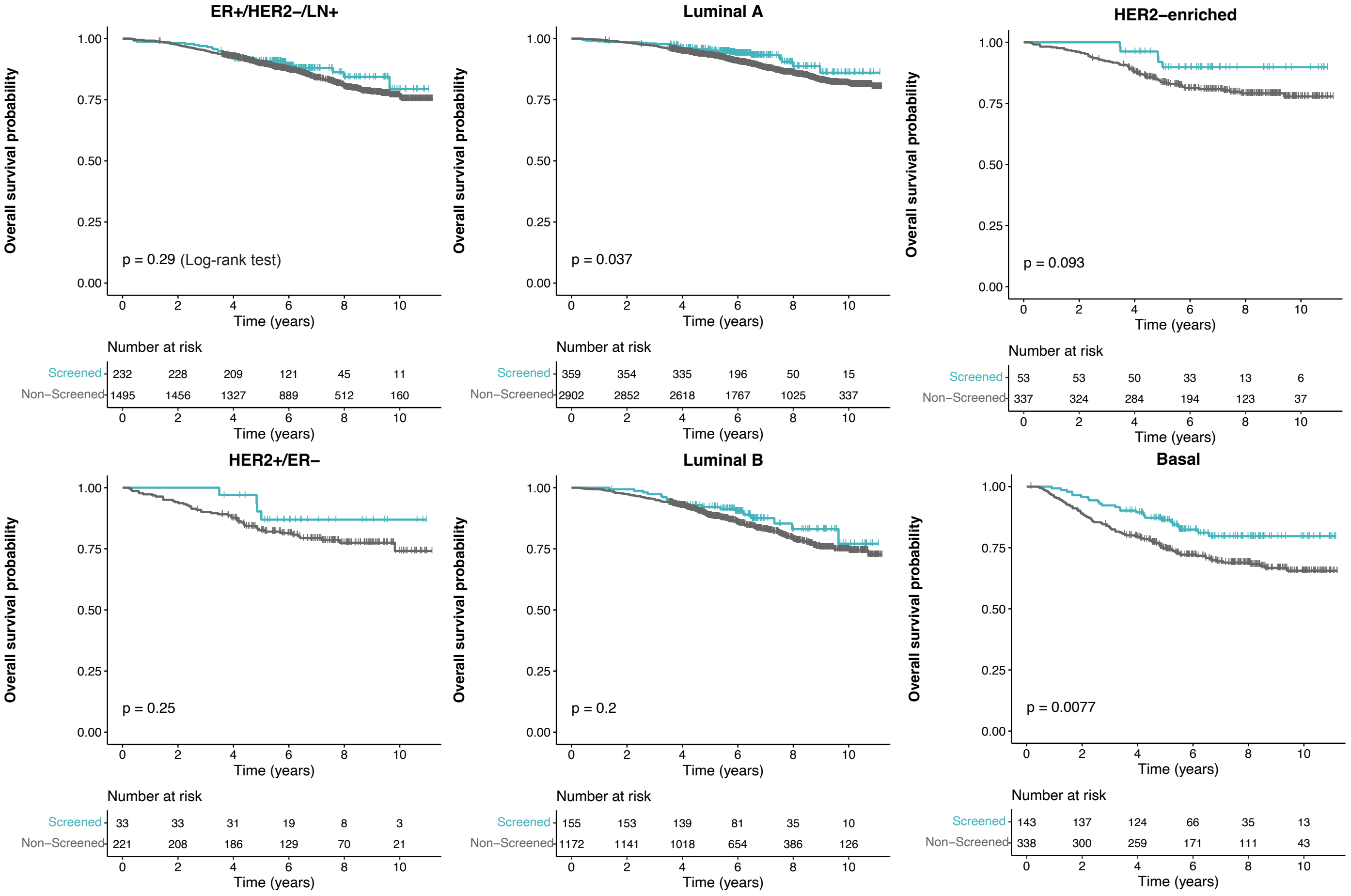

Figure S2. Patient outcome in screening subpopulations. Kaplan-Meier curves using overall survival as endpoint contrasting patients that have and have not been screened for pathogenic germline variants in 11 genes associated with higher risk of breast cancer. Curves include patients in two relevant breast cancer clinical subgroups and four PAM50 molecular subtypes.

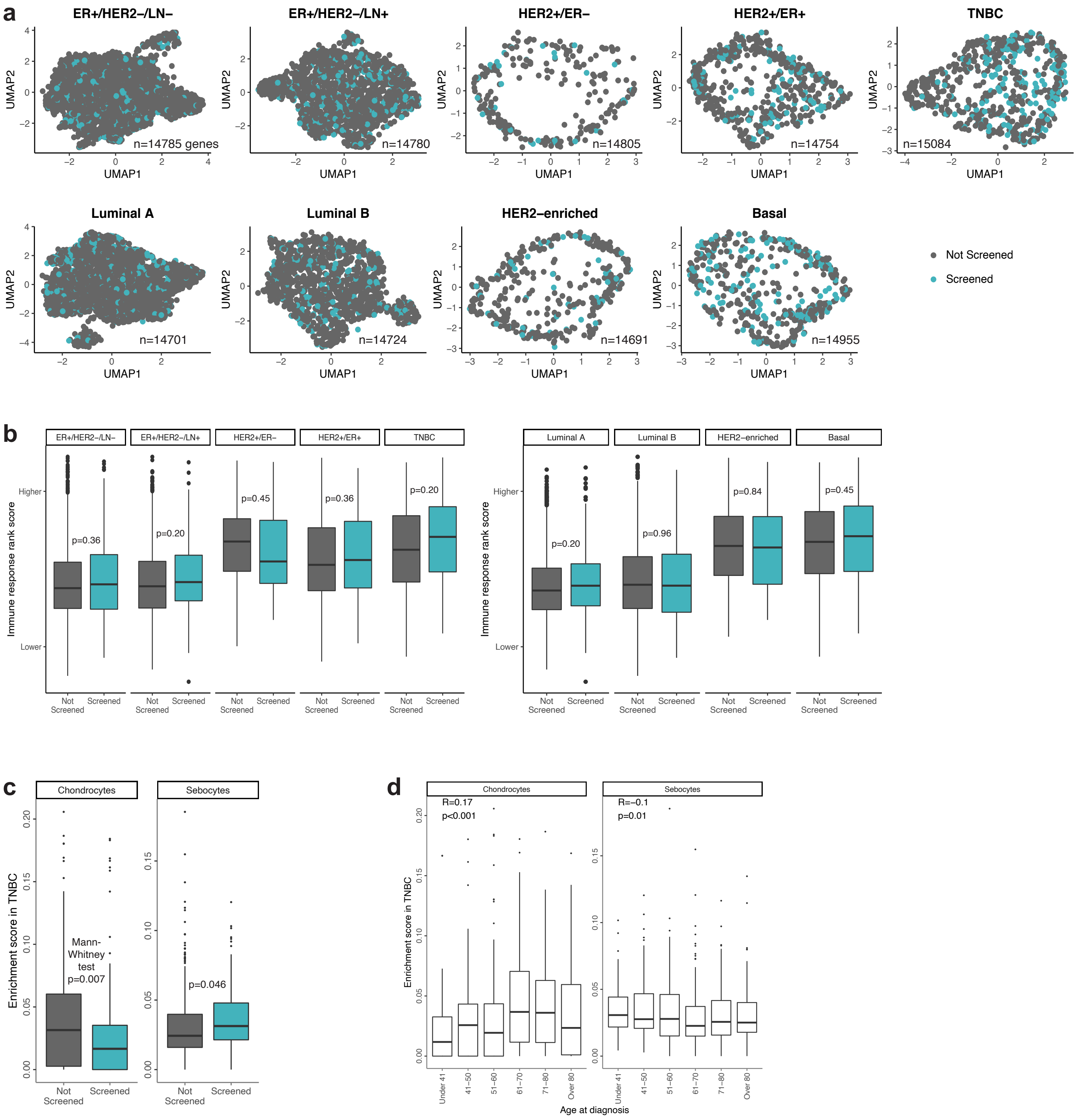

Figure S3. Differences found through gene expression data between screened and non-screened patients within subgroups/subtypes. (a) First two UMAP components calculated using bulk RNA-seq gene expression data of n genes. (b) Distribution of an immune response measure calculated *in silico* by clinical subgroups and PAM50 subtypes. (c) Enrichment score of two cell types by screening status and (d) by age at diagnosis group with all TNBC cases combined. R = Spearman's correlation coefficient.

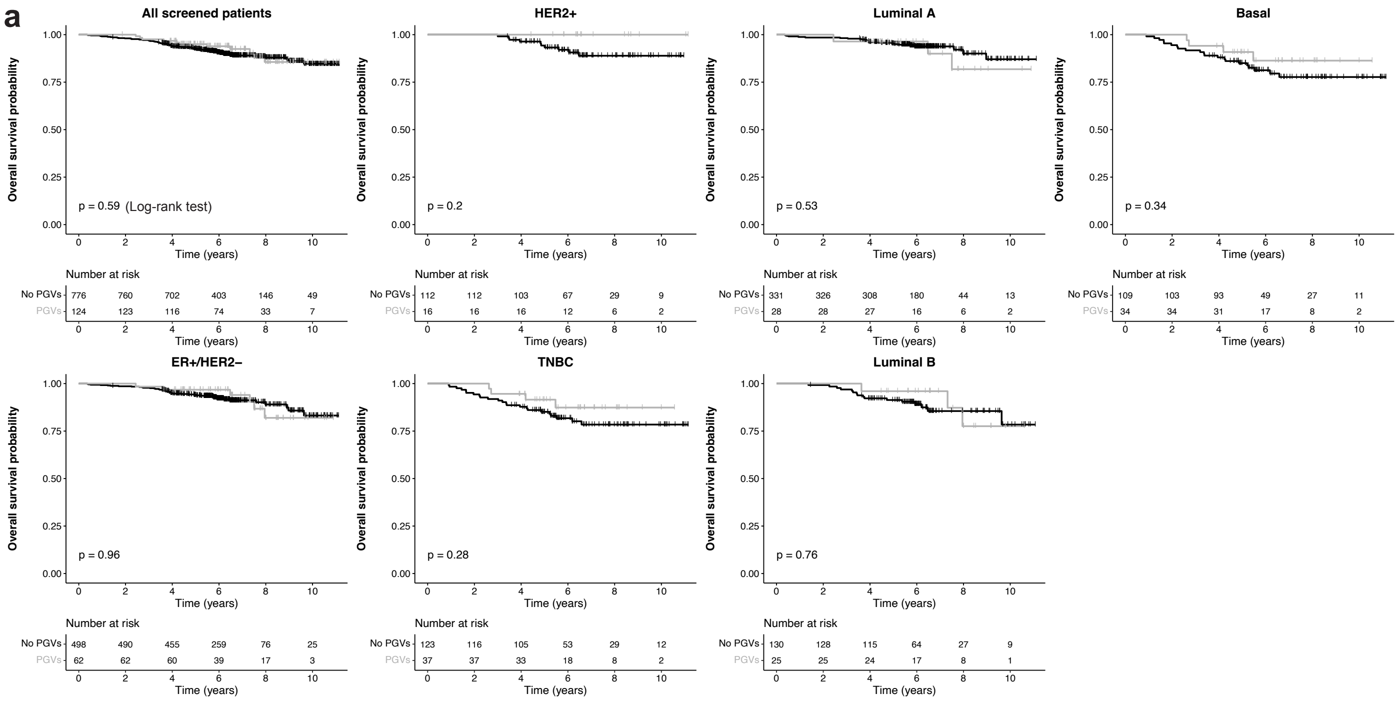

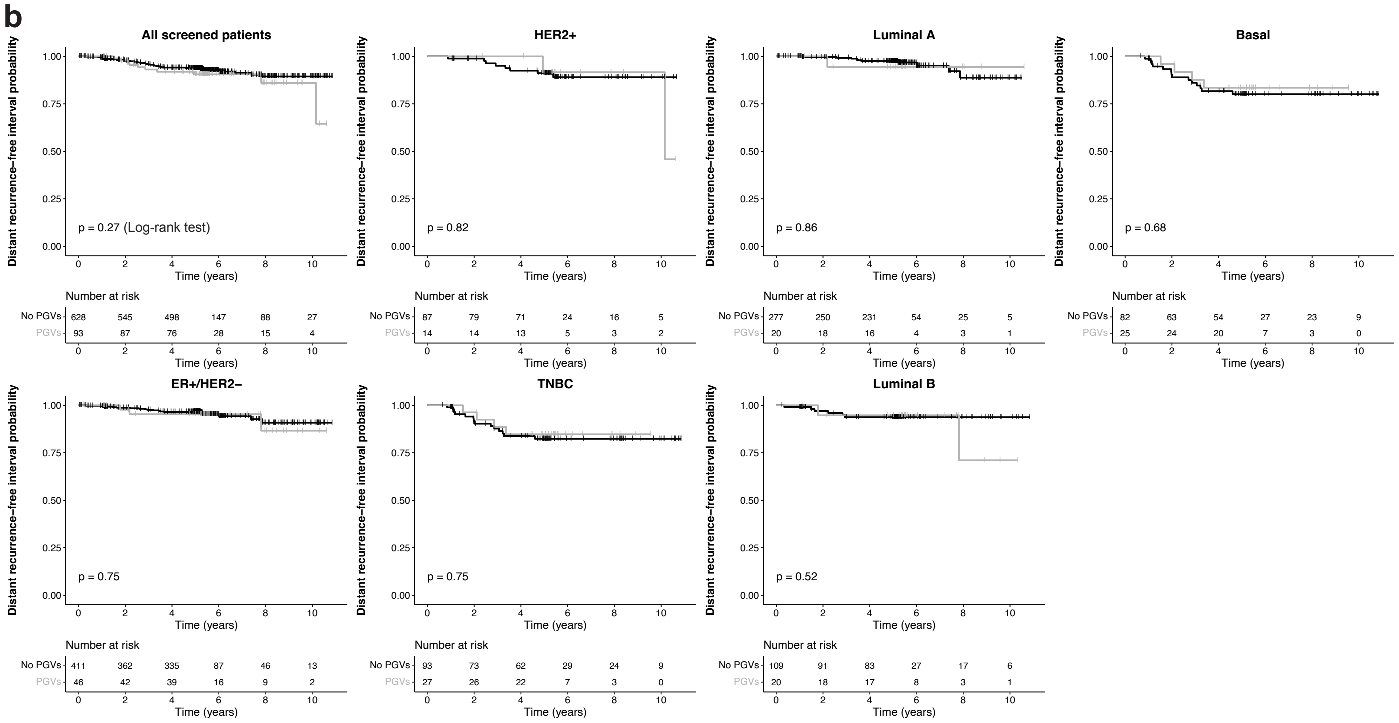

Figure S4. Patient outcome in screened patients with and without pathogenic germline variants (PGVs). Kaplan-Meier curves using (a) overall survival or (b) distant recurrence-free survival as endpoint contrasting screened patients with and without PGVs in any of 11 genes in the the study for all screened patients, three relevant breast cancer clinical subgroups, and three PAM50 molecular subtypes. The PAM50 HER2-enriched subtype had too few samples with PGVs and was not analyzed.

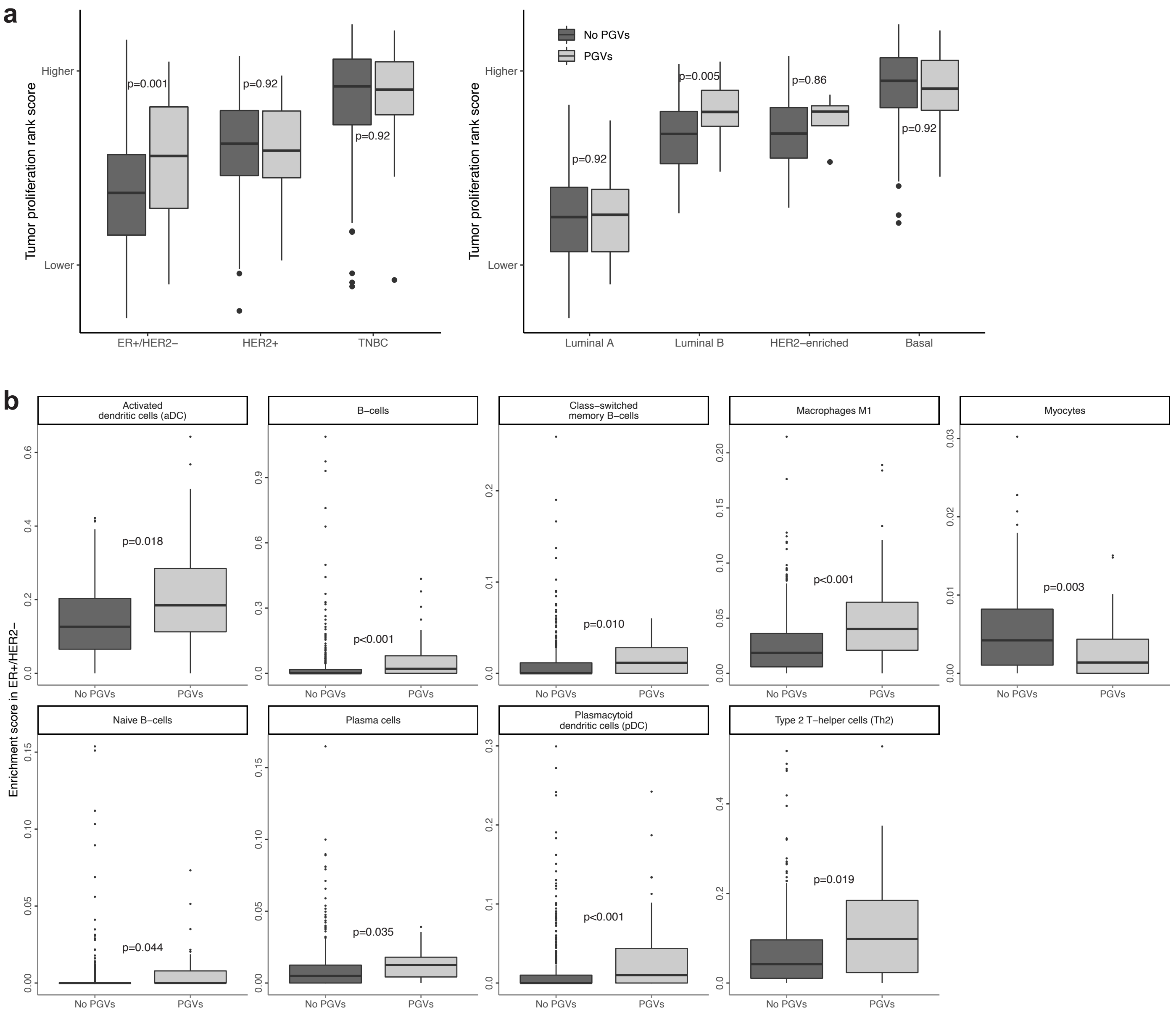

Figure S5. Differences found through gene expression data between screened patients that are carriers of pathogenic germline variants (PGVs) or not within clinical subgroups/PAM50 molecular subtypes. (a) Distribution of a tumor proliferation measure calculated *in silico* per sample by PGV status. (b) Enrichment scores of nine cell types with statistically significant differences between PGV groups calculated by xCell.
